# Cyclic GMP-AMP Ameliorates Diet-induced Metabolic Dysregulation and Regulates Proinflammatory Responses Distinctly from STING Activation

**DOI:** 10.1038/s41598-017-05884-y

**Published:** 2017-07-25

**Authors:** Xin Guo, Chang Shu, Honggui Li, Ya Pei, Shih-Lung Woo, Juan Zheng, Mengyang Liu, Hang Xu, Rachel Botchlett, Ting Guo, Yuli Cai, Xinsheng Gao, Jing Zhou, Lu Chen, Qifu Li, Xiaoqiu Xiao, Linglin Xie, Ke K. Zhang, Jun-Yuan Ji, Yuqing Huo, Fanyin Meng, Gianfranco Alpini, Pingwei Li, Chaodong Wu

**Affiliations:** 1 Department of Nutrition and Food Science, Texas A&M University, College Station, TX 77843 USA; 2 Department of Biochemistry and Biophysics, Texas A&M University, College Station, TX 77843 USA; 3 Department of Molecular and Cellular Medicine, College of Medicine, Texas A&M University Health Science Center, College Station, Texas 77843 USA; 4 Department of Endocrinology and the First Affiliated Hospital of Chongqing Medical University, Chongqing, 400016 China; 5 The Laboratory of Lipid & Glucose Metabolism, the First Affiliated Hospital of Chongqing Medical University, Chongqing, 400016 China; 6 Department of Pathology, School of Medicine and Health Sciences, University of North Dakota, Grand Forks, ND 58202 USA; 7 Vascular Biology Center, Department of Cellular Biology and Anatomy, Medical College of Georgia, Augusta University, Augusta, GA 30912 USA; 8 Drug Discovery Center, Key Laboratory of Chemical Genomics, Peking University Shenzhen Graduate School, Shenzhen, 518055 China; 9 Departments of Medical Physiology and Medicine, Texas A&M University Health Science Center, Temple, TX, 76504, USA

## Abstract

Endogenous cyclic GMP-AMP (cGAMP) binds and activates STING to induce type I interferons. However, whether cGAMP plays any roles in regulating metabolic homeostasis remains unknown. Here we show that exogenous cGAMP ameliorates obesity-associated metabolic dysregulation and uniquely alters proinflammatory responses. In obese mice, treatment with cGAMP significantly decreases diet-induced proinflammatory responses in liver and adipose tissues and ameliorates metabolic dysregulation. Strikingly, cGAMP exerts cell-type-specific anti-inflammatory effects on macrophages, hepatocytes, and adipocytes, which is distinct from the effect of STING activation by DMXAA on enhancing proinflammatory responses. While enhancing insulin-stimulated Akt phosphorylation in hepatocytes and adipocytes, cGAMP weakens the effects of glucagon on stimulating hepatocyte gluconeogenic enzyme expression and glucose output and blunts palmitate-induced hepatocyte fat deposition in an Akt-dependent manner. Taken together, these results suggest an essential role for cGAMP in linking innate immunity and metabolic homeostasis, indicating potential applications of cGAMP in treating obesity-associated inflammatory and metabolic diseases.

## Introduction

Accumulating evidence demonstrates an essential role for the innate immune system in regulating metabolic homeostasis. For instance, activation of the innate immune system has been implicated as a critical factor in development of immunometabolic diseases including diabetes, cardiovascular disease, and non-alcoholic fatty liver disease^1–5^. Considering these critical connections, a better understanding of how the innate immune system interacts with the metabolic system is crucial for uncovering the mysteries of metabolic diseases, and, importantly, for developing novel immunometabolism-based approaches for the prevention and treatment of metabolic diseases.

In the innate immune system, macrophage is one of the most studied cell types from the perspective of metabolic homeostasis^6, 7^. Using approaches involving myeloid cell-specific gene disruption and/or bone marrow transplantation, a number of regulators such as peroxisome proliferator-activated receptor gamma (PPARγ), Jun-N terminal kinase 1 (JNK1), Toll-like receptor 4 (TLR4), Period (Per)1/Per2, and hypoxia-inducible factor (HIF)-2α are shown to alter the inflammatory status of macrophages, which in turn interact with metabolic cells, i.e., hepatocytes and adipocytes, to maintain glucose homeostasis physiologically or to contribute to glucose dysregulation pathologically^8–12^. These findings demonstrate the importance of the innate immune system, in particular macrophages, in controlling metabolic homeostasis. However, it remains largely unknown how regulators of innate immunity modulate functions of metabolic cells in the context of metabolic homeostasis.

Cyclic GMP-AMP (cGAMP) is a novel second messenger in innate immunity that regulates type I interferon (IFN) production^13–18^. In response to cytosolic DNA stimulation, cGAMP synthase (cGAS) catalyzes the synthesis of cGAMP from ATP and GTP^14^. On the one hand, cGAMP binds and activates stimulator of interferon genes (STING), which in turn mediates the activation of TANK-binding kinase 1 (TBK1), thereby stimulating interferon regulatory factor-3 (IRF3)-dependent expression of type I IFNs. Interestingly, cGAMP may subsequently activate negative-feedback regulation of STING activity, thereby preventing the persistent transcription of innate immune genes in dendritic cells^19^. This suggests a role for cGAMP in protecting against inflammation^19^. On the other hand, cGAMP is capable of switching anti-inflammatory macrophages (M2) back to proinflammatory activation (M1)^20^, suggesting a proinflammatory activity of cGAMP. To date, the bio-pathophysiological roles for cGAMP in regulating metabolic homeostasis are unexplored. In particular, it is unknown whether cGAMP is capable of altering functions of metabolic cells, i.e., hepatocytes and adipocytes, and, consequently, whole body glucose metabolic homeostasis. Here we summarize our analyses suggesting novel and essential roles of exogenous cGAMP in regulating innate immunity and metabolic homeostasis.

## Materials and Methods

### cGAMP

cGAMP was synthesized using recombinant cGAS and purified through ultrafiltration followed by anion exchange chromatography. The purified cGAMP was characterized using LC/MS and NMR, and showed purity greater than 95% with endotoxin levels less than 0.005 EU/μg. Chemically synthesized cGAMP (Cat. Code: tlrl-nacga23–5) was purchased from InvivoGen (San Diego, CA, USA).

### Animal experiments

Wild-type (WT) C57BL/6 J mice and STING-disrupted (STING^gt^) mice (C57BL/6 J background) were obtained from the Jackson Laboratory and maintained on a 12:12-h light-dark cycle (lights on at 06:00). For Study 1, male C57BL/6 J mice, at 12 weeks of age, were injected with cGAMP (1 mg/kg, dissolved in phosphate-buffered saline (PBS)) via the tail vein. Blood samples were collected before and at various time points post cGAMP injection to examine cGAMP kinetics. For Study 2, male C57BL/6 J mice, at 5–6 weeks of age, were fed a high-fat diet (HFD) (60% fat calories, 20% protein calories, and 20% carbohydrate calories) or low-fat diet (LFD) (10% fat calories, 20% protein calories, and 70% carbohydrate calories) for 12 weeks. During the last 4 weeks of feeding period, HFD-fed mice were treated with cGAMP (0.2 mg/kg/d, in PBS, intraperitoneally). Some HFD-fed mice and LFD-fed mice were given intraperitoneal injections of PBS and served as controls. After the feeding/treatment period, mice were fasted for 4 hr before sacrifice for collection of blood and tissue samples. Abdominal fat content and liver weight were recorded. Also, liver and adipose tissues were either fixed and embedded for histological and immunohistochemical analyses or frozen stored at −80 °C for further analyses. Some mice were subjected to glucose and insulin tolerance tests and insulin signaling analyses as previously described^21–23^. All study protocols were approved by the Institutional Animal Care and Use Committee of Texas A&M University. In addition, all experiments were performed in accordance with relevant guidelines and regulations.

### Isolation of adipose tissue stromal vascular cells and flow cytometry

Adipose tissue stromal vascular cells (SVC) were isolated using the collagenase digestion method^24, 25^. The isolated SVC were subjected to FACS analyses^11, 26^.

### Plasma parameters

For Study 1, plasma cGAMP levels were measured using a competitive radioimmunoassay (RIA). Briefly, each individual plasma sample was mixed with ^32^P-labeled cGAMP and added to a 96-well plate that was pre-coated with streptavidin and pre-incubated with recombinant human STING. After incubation and wash, the radioactivity of each tested well was counted using scintillation counter. For Studies 1 and 2, plasma IFNβ levels were measured using an ELISA kit. For study 2, plasma glucose and insulin levels were measured using a metabolic assay kit and an ELISA kit (Crystal Chem Inc., Downers Grove, IL), respectively.

### Histological and immunohistochemical analyses

The paraffin-embedded tissue blocks were cut into sections of 5 µm thickness and stained with H&E and/or stained for the expression of F4/80 with rabbit anti-F4/80 antibodies (1:100) (AbD Serotec, Raleigh, NC).

### Cell culture and treatment

Bone marrow cells were isolated from WT mice and STING^gt^ mice and differentiated into macrophages (BMDM) as previously described^8, 27^. After differentiation, both WT BMDM and STING^gt^ BMDM were treated with or without cGAMP (20 µg/ml) for 24 hr in the presence or absence of lipopolysaccharide (LPS) (20 ng/ml) for the last 6 hr. In parallel, both WT BMDM and STING^gt^ BMDM were treated with commercial cGAMP at a dose of 20 µg/ml for 0, 6, 24, and/or 48 hr or at a dose of 0, 5, 20 and/or 40 µg/ml for 24 hr. BMDM-conditioned media were measured for IFNβ levels. Additionally, some WT BMDM were treated with cGAMP (20 µg/ml) for 24 hr in the presence or absence of LPS (100 ng/ml) for the last 30 min to analyze inflammatory signaling or LPS (20 ng/ml) for the last 6 hr to analyze cytokine expression. For hepatocyte studies, primary hepatocytes were isolated from WT mice^28^ and treated with cGAMP (20 µg/ml) or PBS for 24 hr in the presence or absence of LPS (20 ng/ml) for the last 6 hr to quantify the mRNA levels of proinflammatory cytokines and metabolic enzymes. Some hepatocytes were treated with cGAMP similarly and incubated with or without LPS (100 ng/ml) or insulin (100 nM) for the 30 min prior to harvest to examine inflammatory signaling and insulin signaling, respectively. Additional primary hepatocytes were used for glucose production measurement, the reporter assay, and fat deposition assessment as described below. For adipocyte studies, 3T3-L1 cells were maintained in high glucose DMEM and differentiated as previously described^21, 22^. After differentiation, adipocytes were treated with cGAMP to examine adipocyte response. To examine whether cGAMP treatment and STING activation generate similar effects on the inflammatory responses, BMDM, primary hepatocytes, and/or adipocytes were also treated with or without 5,6-dimethylxanthenone-4-acetic acid (DMXAA, InvivoGen, Cat. Code: tlrl-dmx), a known activator of mouse STING^27^, at a dose of 75 µg/ml for 1 hr or 6 hr in the presence or not of LPS (100 ng/ml) for the last 30 min.

### Molecular assays

To assess inflammatory and/or insulin signaling, lysates of frozen tissues and/or cultured cells were subjected to Western blot analysis as previously described^29, 30^. The maximum intensity of each band was quantified using ImageJ software. Ratios of Pp46/p46, Pp65/p65, PAkt/Akt, and PTBK1/TBK1 were normalized to GAPDH and adjusted relative to the average of PBS-treated control or LFD-PBS, which was arbitrarily set as 1 (AU). To examine gene expression, the total RNA was isolated from frozen tissue samples and cultured/isolated cells, and subjected to reverse transcription and real-time PCR analysis. Results were normalized to 18 s ribosomal RNA and plotted as relative expression to the average of PBS-treated control, which was set as 1. Also, liver RNA samples prepared from Study 2 were subjected to OneArray® to profile differentially expressed genes (PhalanxBio Inc., San Diego, CA).

### Hepatocyte glucose output (HGO)

WT primary mouse hepatocytes were treated with cGAMP (20 µg/ml) or PBS for 24 hr in the presence or absence of an Akt inhibitor (MK-2206, 1 µM) or a TBK1 inhibitor (BX795, 1 µM). The cells were then switched to glucose-free media supplemented with sodium lactate (20 mM) and sodium pyruvate (2 mM)) and treated with or without glucagon (100 nM) for an additional 4 hr prior to harvest for measurement of HGO. The latter was calculated by normalizing the concentrations of glucose in the media with protein concentrations of cell lysates.

### The gene promoter activity assay

WT primary mouse hepatocytes were transfected with a plasmid in which luciferase expression was driven by a G6Pase promoter (pG6Pase), a PEPCK promoter (pPEPCK), or an empty promoter sequence (pGL3) using Lipofectamine Plus (Invitrogen) according to the manufacturer’s instructions. After transection and incubation with normal M199 media for 24 hr, the transfected cells were treated with cGAMP (20 µg/ml) or PBS for an additional 24 hr in the presence or absence of an Akt inhibitor (MK-2206, 1 µM) or a TBK1 inhibitor (BX795, 1 µM). Thereafter, the cells were treated with or without glucagon (100 nM) for 4 hr and harvested for determination of luciferase activity using a Dual Luciferase Reporter Assay System (Promega, Madison, WI). The promoter transcription activity was calculated by normalizing the luciferase activity with protein concentrations and was expressed as arbitrary unit (AU).

### Hepatocyte fat deposition

WT primary mouse hepatocytes were treated with cGAMP (20 µg/ml) or PBS for 24 hr in the presence or absence of an Akt inhibitor (MK-2206, 1 µM). Concurrently, the cells were treated with or without palmitate (250 µM) for 24 hr. At 1 hr prior to harvest, the cells were stained with Oil-Red-O.

### Statistical Methods and Microarray Data Analysis

Numeric data are presented as means ± SE (standard error). Statistical significance was assessed by unpaired, two-tailed ANOVA and/or Student’s *t* tests. Differences were considered significant at the two-tailed *P* < 0.05. Microarray data analysis was performed as previously described^31–33^.

## Results

### Exogenous cGAMP stimulates IFNβ production in wild-type macrophages and mice

**Figure 1:**
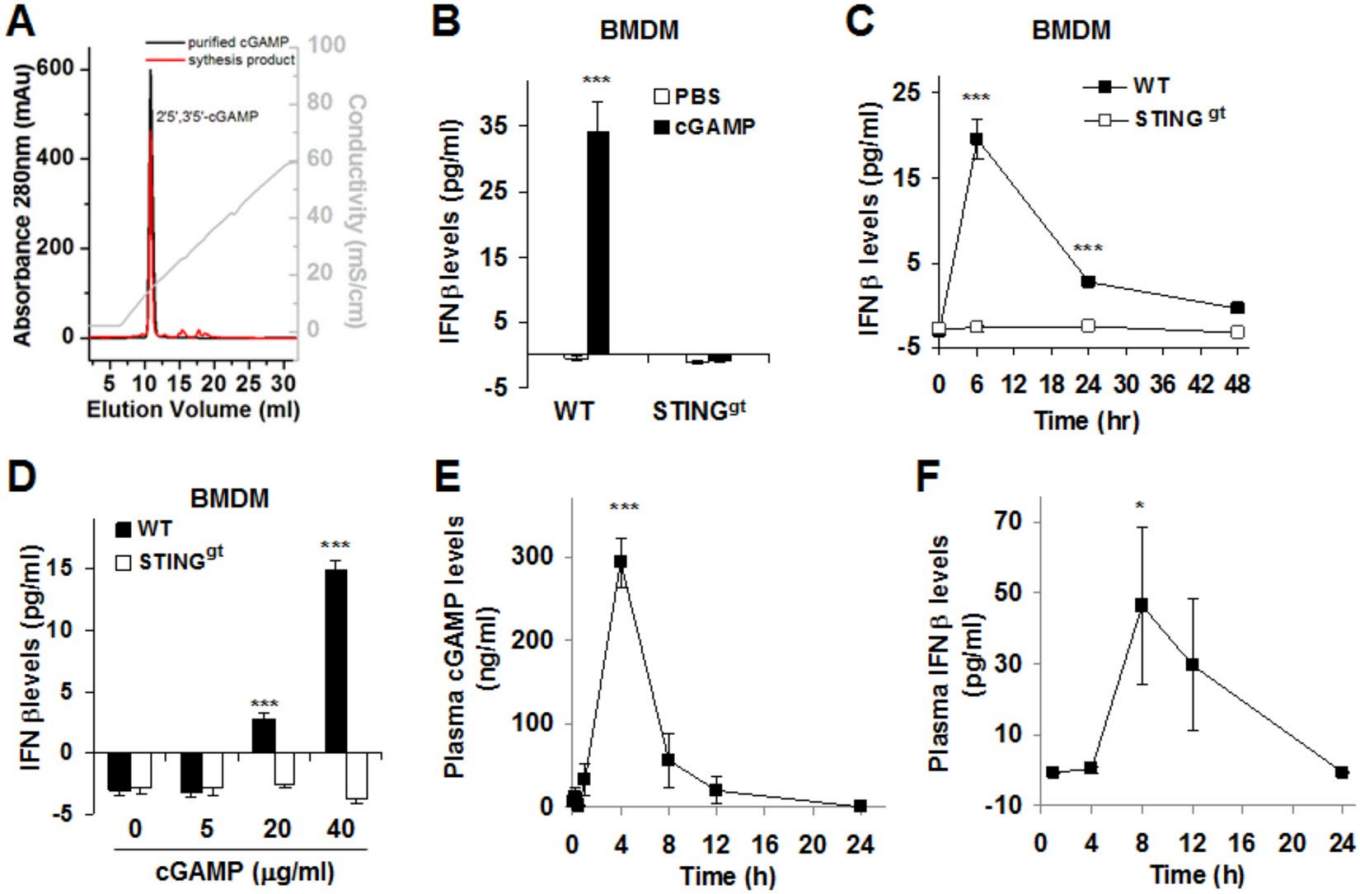
Exogenous cGAMP stimulates interferon beta production from macrophages and mice. (**A**) Enzymatically synthesized and purified cGAMP. (**B**) Effects of synthesized (exogenous) cGAMP on macrophage interferon beta (IFNβ) production. (**C**) Time-course study of cGAMP stimulation of macrophage IFNβ production. (**D**) Dose-response study of cGAMP stimulation of macrophage IFNβ production. (**E**) Kinetics of exogenous cGAMP in C57BL/6 J mice. (**F**) Effects of exogenous cGAMP on plasma levels of IFNβ in C57BL/6 J mice. For B–D, bone marrow cells were isolated from male wild-type (WT) C57BL/6 J mice and STING-disrupted (STING^gt^) mice, and differentiated into macrophages (BMDM). After differentiation, BMDM were treated with or without enzymatically synthesized cGAMP (20 µg/ml in B), commercial chemically synthesized cGAMP (20 µg/ml in C or the indicated doses in D), or PBS for 24 hr (B and D) or the indicated time periods (C). For E and F, chow diet-fed male C57BL/6 J mice, at 12 weeks of age, were injected with cGAMP (1 mg/kg, dissolved in PBS) via the tail vein. Blood samples were collected at the given time points and used to quantify plasma levels of cGAMP (E) and IFNβ (F). For B–F, data are means ± S.E. n = 6. *P < 0.05 and ***P < 0.001 cGAMP vs. PBS for WT cells (in B), WT vs. STING^gt^ for the same time point (in C) or dose (in D), or the indicated time point vs. any of the rest time point (in E and F).

Enzymatically synthesized (exogenous) cGAMP (Fig. 1A) was subjected to functional validation. Compared with control, cGAMP treatment markedly stimulated IFNβ production in BMDM from WT C57BL/6 J mice, but not in BMDM from STING^gt^ mice (Fig. 1B). Additionally, the effect of exogenous cGAMP on inducing IFNβ production in WT BMDM was not altered by LPS (Supplemental Figure S1A). Unlike in WT BMDM, cGAMP did not stimulate IFNβ production in primary hepatocytes from either WT or STING^gt^ mice, as well as adipocytes differentiated from 3T3-L1 cells and/or STING^gt^ stromal cells (data not shown). As confirmatory studies, we treated BMDM with commercial chemically synthesized cGAMP. Consistently, commercial cGAMP stimulated IFNβ production in WT BMDM, but not in STING^gt^ BMDM. In the time-course study, cGAMP stimulation of IFNβ production peaked at 6 hr post cGAMP treatment in WT BMDM (Fig. 1C). Within a dose range of 0 to 40 µg/ml, cGAMP stimulation of IFNβ production was dose-dependent (Fig. 1D). To validate a role of STING activation in IFNβ production, we treated BMDM, primary mouse hepatocytes and/or adipocytes with DMXAA. As expected, DMXAA treatment markedly increased IFNβ production in WT BMDM, but not in STING^gt^ BMDM (Supplemental Figure S1B). Similar to the effect of exogenous cGAMP, DMXAA treatment did not stimulate IFNβ production in primary hepatocytes from either WT or STING^gt^ mice. Unlike exogenous cGAMP, DMXAA treatment significantly increased IFNβ production in differentiated 3T3-L1 adipocytes (Supplemental Figure S1B).

We also examined the *in vivo* kinetics and activity of exogenous cGAMP in WT C57BL/6 J mice using a RIA (Supplemental Figure S2). We observed a unique kinetics of cGAMP (Fig. 1E), in which the plasma cGAMP levels peaked at 4 to 6 hr after an intravenous injection of cGAMP. In terms of IFNβ production, cGAMP induced peak plasma IFNβ levels at 8 hr post cGAMP injection (Fig. 1F). Therefore, exogenous cGAMP is active in WT mice.

### Exogenous cGAMP improves systemic glucose homeostasis while decreasing liver TBK1 phosphorylation in HFD-fed C57BL/6 J mice

**Figure 2:**
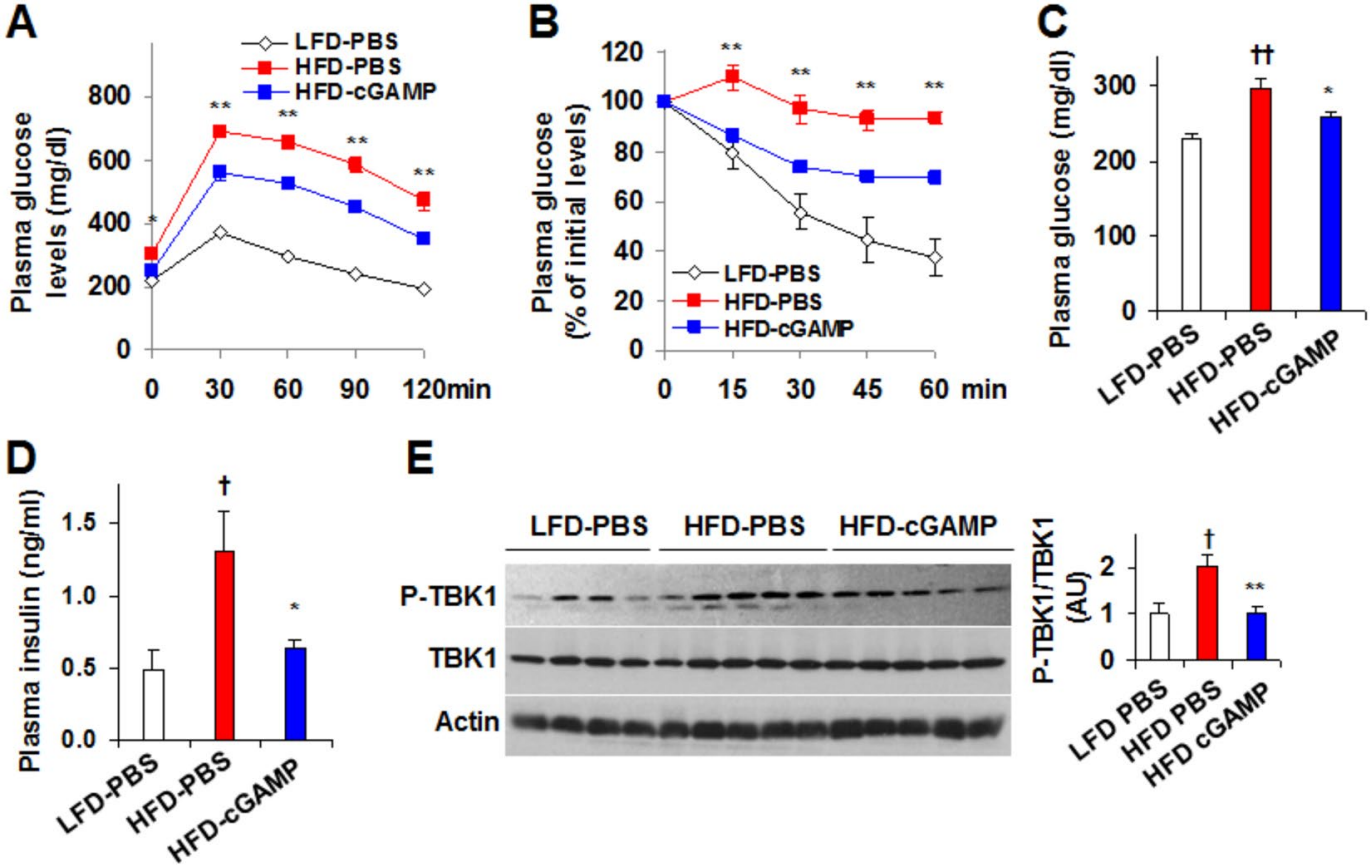
Treatment with cGAMP improves systemic glucose homeostasis and insulin sensitivity while decreasing liver TBK1 phosphorylation in HFD-fed C57BL/6 J mice. (**A**,**B**) Glucose (A) and insulin (B) tolerance tests. (**C**,**D**) Plasma levels of glucose (C) and insulin (D). (**E**) Liver TBK1 phosphorylation. For A–E, male C57BL/6 J mice, at 5–6 weeks of age, were fed a high-fat diet (HFD) for 12 weeks and treated with enzymatically synthesized cGAMP (intraperitoneally, 0.2 mg/kg/d, in PBS) or PBS for the last 4 weeks. Age- and gender-matched mice were fed a low-fat diet (LFD) and treated with PBS. For A and B, after the feeding/treatment period, mice were fasted for 4 hr and subjected to an intraperitoneal injection of glucose (2 g/kg body weight) or insulin (1 U/kg body weight) for glucose (A) and insulin (B) tolerance tests, respectively. For C and D, after 12 week feeding/treatment periods, mice were fasted for 4 hr. Blood samples were collected prior to tissue harvest. Plasma levels of glucose and insulin were measured using a chemical kit and an ELISA kit, respectively. For E, TBK1 phosphorylation was examined using Western blot analysis. Cropped blots were displayed. Also, full-length blots were included in Supplemental Information (Figure S8). AU, arbitrary unit. For A–E, numeric data are means ± S.E. n = 10–12. *P < 0.05 and **P < 0.01 HFD-cGAMP vs. HFD-PBS (C–E) for the same time point (A and B); ^†^ *P* < 0.05 and ^††^ *P* < 0.01 HFD-PBS vs. LFD-PBS (C–E).

Roles of cGAMP in obesity and type 2 diabetes are unknown. Since exogenous cGAMP is active *in vivo*, we decided to examine the metabolic consequence of exogenous cGAMP in mice with diet-induced obesity (DIO), e.g., C57BL/6 J mice upon HFD feeding for 12 weeks. Also, we analyzed liver TBK1 phosphorylation states in DIO mice; considering that an effective assay to quantify tissue cGAMP concentrations is not available and that TBK1 is activated upon endogenous cGAMP binding and activating STING. Compared with those in LFD-fed and PBS-treated (LFD-PBS) mice, HFD-fed and PBS-treated (HFD-PBS) mice were glucose intolerant and displayed overt systemic insulin resistance, hyperglycemia, and hyperinsulinemia (Fig. 2A–D). Additionally, HFD-PBS mice displayed a significant increase in liver TBK1 phosphorylation states compared with LFD-PBS mice (Fig. 2E). Among HFD-fed mice, cGAMP treatment significantly reduced liver TBK1 phosphorylation states compared with control (Fig. 2E), which was accompanied with improvement of systemic metabolism. The latter was evidenced by the finding that HFD-fed and cGAMP-treated (HFD-cGAMP) mice displayed significant decreases in the severity of systemic glucose intolerance, insulin resistance, hyperglycemia, and/or hyperinsulinemia compared with HFD-PBS mice (Fig. 2A–D). Compared with control, cGAMP treatment did not significantly alter body weight and food intake (Supplemental Figure S3). In addition, the plasma IFNβ levels were nearly undetectable in all mice (data not shown). Taken together, these results indicate that the enhanced liver TBK1 phosphorylation is positively correlated with obesity-associated glucose dysregulation and insulin resistance. Furthermore, reducing liver STING/TBK1 signaling may contribute to the effect of cGAMP on improving hepatic metabolic responses, as well as systemic glucose dysregulation. We also examined the phosphorylation states of liver IRF3, which is phosphorylated and activated by TBK1^34^. However, we did not observe any statistical differences upon cGAMP treatment (data not shown).

### Treatment with cGAMP ameliorates diet-induced proinflammatory responses in both the liver and adipose tissue of C57BL/6 J mice

**Figure 3:**
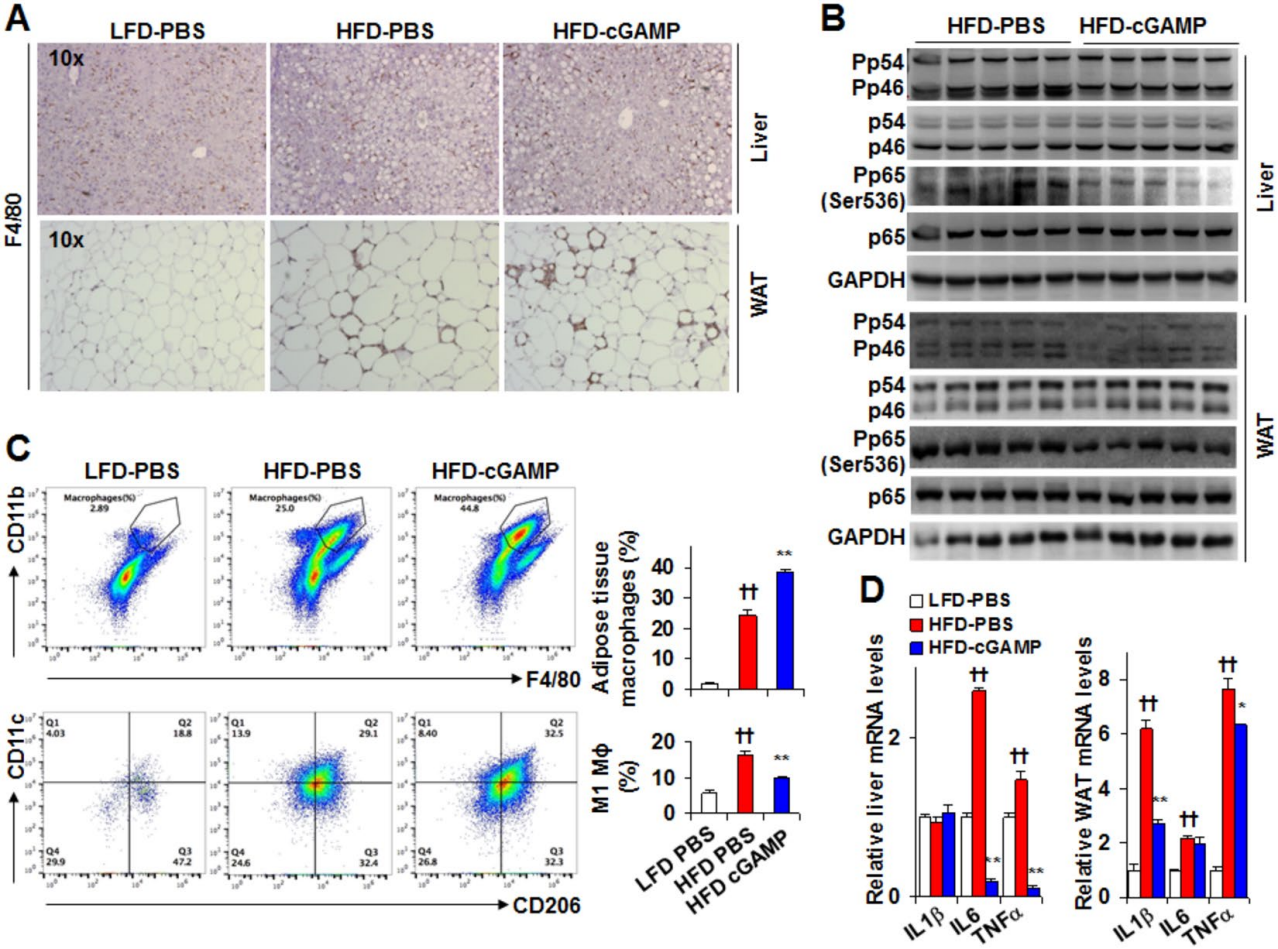
Treatment with cGAMP ameliorates diet-induced liver and adipose tissue inflammation. (**A**) Liver (top) and adipose tissue (bottom) sections were stained for F4/80. (**B**) Liver and adipose tissue proinflammatory signaling was examined using Western blot analysis. Cropped blots were displayed. Also, full-length blots were included in Supplemental Information (Figures S9 and S10). (**C**) Adipose tissue macrophage infiltration and polarization. (**D**) Liver and adipose tissue mRNA levels. WAT, white adipose tissue. For A–D, male C57BL/6 J mice, at 5–6 weeks of age, were fed a high-fat diet (HFD) for 12 weeks and treated with cGAMP (intraperitoneally, 0.2 mg/kg/d, in PBS) or PBS for the last 4 weeks. Age- and gender-matched C57BL/6 J mice were fed a low-fat diet (LFD) and treated with PBS. After the feeding/treatment period, mice were fasted for 4 hr prior to harvest. For C, stromal vascular cells (SVC) were isolated from adipose tissue and analyzed for CD11b and F4/80 expression (maturate macrophages). Mature macrophages were further analyzed for CD11c and CD206 expression. Top three panels, representative plots of mature macrophages (F4/80^+^ CD11b^+^ cells); bottom three panels, representative plots of macrophage polarization; top bar graph, percentages of mature macrophages; bottom bar graphs, percentages of M1 macrophages. For bar graphs (in C and D), data are means ± S.E. n = 6–10. *P < 0.05 and **P < 0.01 HFD-cGAMP vs. HFD-PBS (in C) for the same gene (in D); ^††^ *P* < 0.01 HFD-PBS vs. LFD-PBS (in C) for the same gene (in D).

Obesity induces overt liver and adipose tissue inflammation, thereby contributing to systemic metabolic dysregulation^24, 29, 30, 35–38^. We examined the effect of exogenous cGAMP on liver and adipose tissue proinflammatory responses in DIO mice. Upon staining liver sections for F4/80 expression, the numbers of macrophages/Kupffer cells in livers of HFD-cGAMP mice did not differ from those of HFD-PBS mice (Fig. 3A). However, HFD-cGAMP mice exhibited decreases in liver JNK p46 phosphorylation states (0.5-fold) (Fig. 3B and Supplemental Figure S4A) and IL-6 and TNFα mRNAs (Fig. 3D), indicating a significant decrease in liver proinflammatory responses. Similarly, HFD-cGAMP mice displayed significant decreases in adipose tissue JNK p46 (0.5-fold) and NF-κB p65 (0.4-fold) phosphorylation states compared with HFD-PBS mice (Fig. 3B and Supplemental Figure S4B) while adipose tissue of HFD-cGAMP mice contained more F4/80 positive cells (Fig. 3A). Since macrophages critically control adipose tissue inflammatory status^2, 8, 11, 24^, we performed FACS analysis for adipose tissue SVC, the immune cell-containing fraction of collagenase-digested epididymal fat. Compared with PBS, cGAMP treatment increased adipose tissue content of mature macrophages (F4/80^+^ CD11b^+^ cells) in HFD mice (Fig. 3C). However, cGAMP treatment significantly decreased percentages of M1 macrophages (F4/80^+^ CD11b^+^ CD11c^+^ CD206^–^ cells) among SVC without altering percentages of M2 macrophages (Fig. 3C). Additionally, adipose tissue IL-1β and TNFα mRNAs in HFD-cGAMP mice were decreased compared with their respective levels in HFD-PBS mice (Fig. 3D). Taken together, these results suggest that cGAMP treatment ameliorates obesity-associated proinflammatory responses in both the liver and adipose tissue of C57BL/6 J mice.

### Treatment with cGAMP ameliorates diet-induced metabolic dysregulation in both the liver and adipose tissue of C57BL/6 J mice

**Figure 4:**
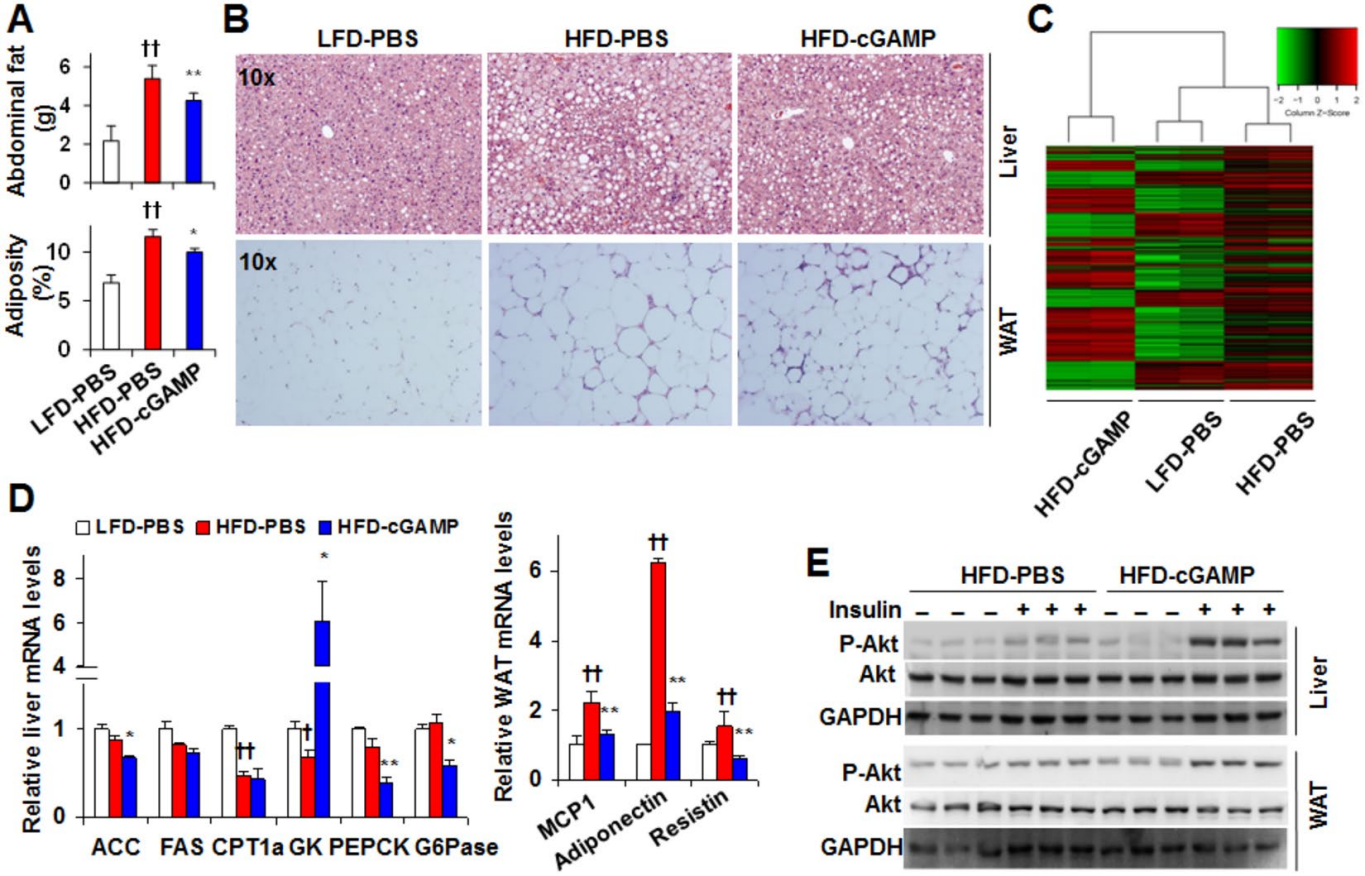
Treatment with cGAMP ameliorates diet-induced liver and adipose tissue metabolic dysregulation. (**A**) Abdominal fat mass and adiposity. (**B**) Liver (top) and adipose tissue (bottom) sections were stained with H&E. (**C**) Liver microarray heat map and sample clustering dendrogram of RNA expression. (**D**) Liver and adipose tissue mRNA levels were analyzed using real-time PCR. (**E**) Liver and adipose tissue insulin signaling. For A–E, male C57BL/6 J mice, at 5–6 weeks of age, were fed a high-fat diet (HFD) for 12 weeks and treated with cGAMP (intraperitoneally, 0.2 mg/kg/d, in PBS) or PBS for the last 4 weeks. Age- and gender-matched C57BL/6 J mice were fed a low-fat diet (LFD) and treated with PBS. After the feeding/treatment periods, mice were fasted for 4 hr prior to harvest. WAT, white adipose tissue. For E, prior to tissue harvest, mice were injected with or without insulin (1 U/kg) into the portal vein for 5 min. Tissue lysates were subjected to Western blot analyses. Cropped blots were displayed. Also, full-length blots were included in Supplemental Information (Figure S11). For bar graphs, data are means ± S.E. n = 6–10. *P < 0.05 and **P < 0.01 HFD-cGAMP vs. HFD-PBS (in A) for the same gene (in D); ^†^ *P* < 0.05 and ^††^ *P* < 0.01 HFD-PBS vs. LFD-PBS (in A) for the same gene (in D).

We examined the effect of exogenous cGAMP on liver and adipose tissue metabolic responses in HFD-fed C57BL/6 J mice. Compared with LFD-PBS mice, HFD-PBS mice displayed significant increases in abdominal fat mass and adiposity (Fig. 4A). Consistently, HFD-PBS mice displayed severe hepatic steatosis and a significant increase in adipocyte size (Fig. 4B). These changes were ameliorated significantly upon cGAMP treatment (Fig. 4A,B). With regard to glucose and fat metabolism, HFD-cGAMP mice were distinguished from controls by changes in liver expression profile of the pertinent genes (Fig. 4C, Supplemental Figure S5 and Table 1). Specifically, HFD-cGAMP mice exhibited significant decreases in liver mRNA levels of phosphoenolpyruvate carboxykinase (PEPCK) and glucose-6-phosphatase (G6Pase), two gluconeogenic enzymes, and a significant increase in the mRNA levels of glucokinase (GK) (Fig. 4D), a glycolytic enzyme. These results were consistent with improved glucose homeostasis. While ameliorating hepatic steatosis, cGAMP treatment significantly decreased the mRNA levels of liver acetyl-CoA carboxylase (ACC), an essential lipogenic enzyme, and an insignificant decrease in the mRNA levels of liver fatty acid synthase (FAS), also a lipogenic enzyme. Additionally, cGAMP treatment did not significantly alter the mRNA levels of liver carnitine palmitoyltransferase (CPT1a) (Fig. 4D), an enzyme that transfers acyl-CoA into mitochondrial matrix for oxidation. With regard to adipose tissue gene expression, cGAMP treatment decreased the mRNA levels of adiponectin, resistin, and monocyte chemoattractant protein-1 (MCP1) (Fig. 4D). When tissue insulin sensitivity was analyzed, insulin signaling, as indicated by insulin-induced Akt (Ser473) phosphorylation, was significantly increased in both the liver and adipose tissue of HFD-cGAMP mice compared with that of HFD-PBS mice (Fig. 4E). Taken together, these results indicate that cGAMP treatment ameliorates diet-induced metabolic dysregulation and insulin resistance in both the liver and adipose tissue.

**Table 1:** cGAMP regulation of liver expression of genes related to metabolic and inflammatory responses.

Gene Symbol	Description	log2(Ratio)		P-value	
		G/H	H/L	G/H	H/L
Hk2	hexokinase 2	1.572	0.646	5.51E-10	4.78E-03
Hk3	hexokinase 3	1.823	0.157	5.12E-10	5.79E-01
Aldoc	aldolase C, fructose bisphosphate	1.138	−0.549	1.88E-04	4.11E-02
Pfkm	phosphofructokinase, muscle	1.54	−0.587	3.09E-10	1.32E-02
G6pc	glucose-6-phosphatase, catalytic	−1.12	−1.323	5.59E-08	3.26E-09
Pck1	Phosphoenolpyruvate carboxykinase 1, cytosolic	−0.456	0.044	1.37E-02	8.04E-01
Ppargc1a	peroxisome proliferative activated receptor, gamma, coactivator 1 alpha	−1.223	−0.479	6.22E-05	1.06E-01
Gys1	glycogen synthase 1, muscle	1.36	0.847	3.43E-09	8.45E-04
Acaca	acetyl-Coenzyme A carboxylase alpha	−0.534	−1.408	2.08E-02	2.78E-07
Acsl3	acyl-CoA synthetase long-chain family member 3	−1.469	−0.263	4.44E-09	1.69E-01
Cpt1b	carnitine palmitoyltransferase 1b	1.218	0.057	2.51E-06	8.54E-01
Ppargc1b	peroxisome proliferative activated receptor, gamma, coactivator 1 beta	0.709	−0.138	2.18E-04	4.68E-01
Ppard	peroxisome proliferator activator receptor delta	0.948	−0.128	2.14E-06	5.31E-01
Ccl25	chemokine (C-C motif) ligand 25	−1.628	0.001	1.41E-06	9.97E-01
Ikbkb	inhibitor of kappaB kinase beta	−1.037	−0.464	5.46E-07	2.30E-02

Male C57BL/6 J mice, at 5–6 weeks of age, were fed a high-fat diet (HFD) for 12 weeks and treated with cGAMP (0.2 mg/kg/d) or PBS for the last 4 weeks. Age- and gender-matched mice were fed a low-fat diet (LFD) and treated with PBS. After the feeding/treatment period, liver samples were subjected to microarray analysis. G/H, HFD-cGAMP/HFD-PBS; H/L, HFD-PBS/LFD-PBS.

### Exogenous cGAMP exerts cell-type-specific anti-inflammatory effects distinctly from STING activation

**Figure 5:**
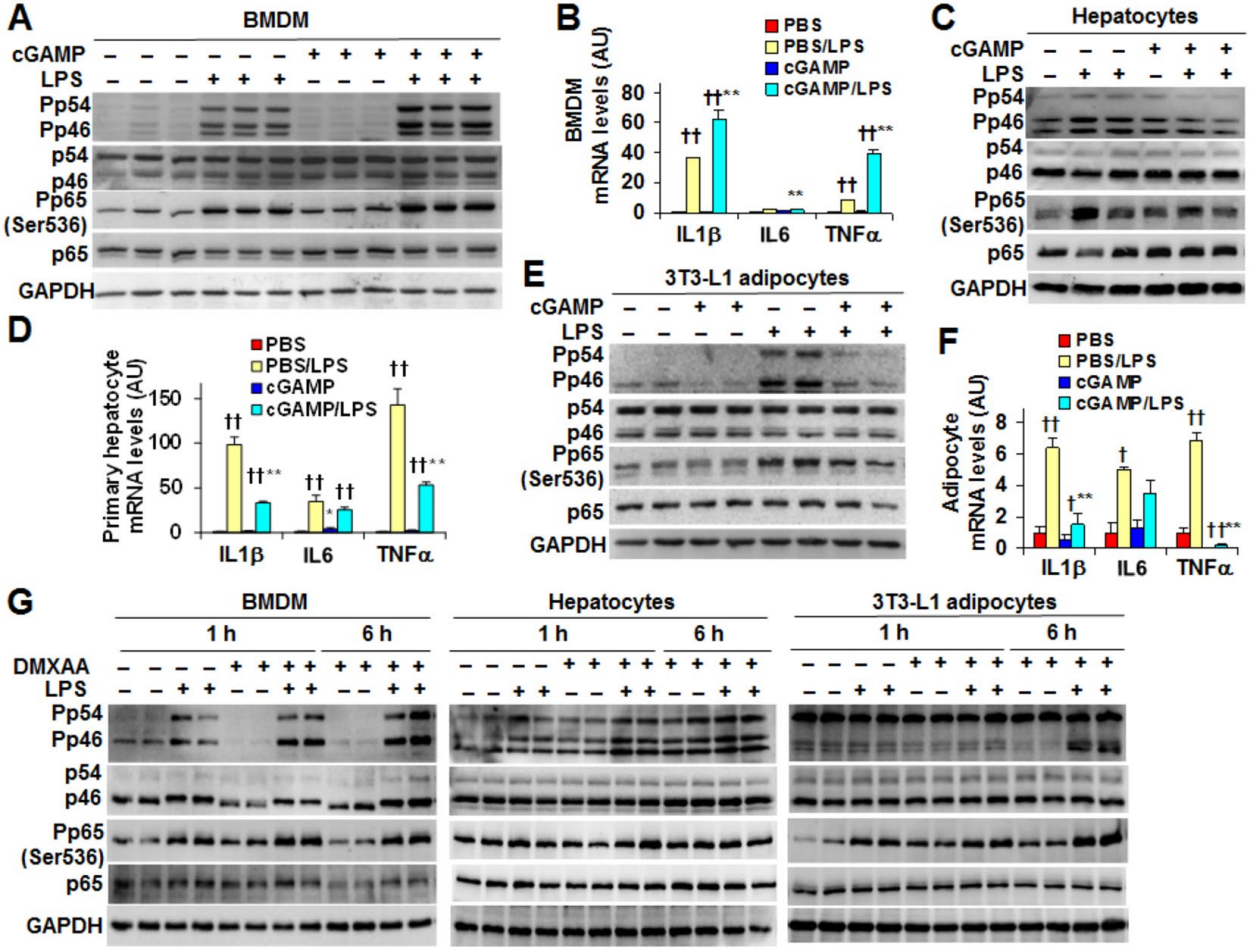
Exogenous cGAMP exerts cell-type-specific anti-inflammatory effects distinctly from STING activation. (**A**,**B**) Proinflammatory signaling (A) and cytokine expression (B) in bone marrow-derived macrophages (BMDM). (**C**,**D**) Proinflammatory signaling (C) and cytokine expression (D) in primary mouse hepatocytes. (**E**,**F**) Proinflammatory signaling (E) and cytokine expression (F) in differentiated 3T3-L1 adipocytes. (**G**) DMXAA, an STING activator, enhances the proinflammatory signaling of macrophages, hepatocytes, and adipocytes. For A–G, bone marrow cells and primary hepatocytes were isolated from male C57BL/6 J mice. Also, bone marrow cells were differentiated into macrophages and 3T3-L1 cells were differentiated into adipocytes prior to cGAMP treatment. For A–F, cells were treated with enzymatically synthesized cGAMP (20 µg/ml in A, B, E, and F), commercial chemically synthesized cGAMP (20 µg/ml in C and D), or PBS for 24 hr in the presence or absence of LPS (100 ng/ml for the last 30 min in A, C, and E; 20 ng/ml for the last 6 hr in B, D, and F). For G, cells were treated with DMXAA (75 µg/ml, 100 × stock in 7.5% NaHCO_3_) or NaHCO_3_ solution (Ctrl) for 1 or 6 hr in the presence or absence of LPS (100 ng/ml) for the last 30 min. For bar graphs, data are means ± S.E. n = 6–8. *, *P* < 0.05 and **P < 0.01 cGAMP vs. PBS or cGAMP/LPS vs. PBS/LPS for the same gene; ^††^ *P* < 0.01 PBS/LPS vs. PBS or cGAMP/LPS vs. cGAMP for the same gene. For A, C, E, and G, cropped blots were displayed. Also, full-length blots were included in Supplemental Information (Figures S12–S17).

The direct effects of exogenous cGAMP on inflammatory responses are unknown. We treated WT BMDM with enzymatically synthesized cGAMP and observed significant increases in LPS-stimulated proinflammatory signaling through JNK p46 and NF-κB p65 (Fig. 5A and Supplemental Figure S6) and expression of IL-1β, IL-6, and TNFα (Fig. 5B). Similar results were obtained in WT BMDM upon treatment with commercial cGAMP (Supplemental Figure S7). Also, we observed that cyclic AMP did not alter WT BMDM responses as did cGAMP (data not shown). These results suggest that exogenous cGAMP is capable of enhancing the proinflammatory activation of cultured WT macrophages. Unlike in BMDM, cGAMP treatment displayed anti-inflammatory effects in both WT primary mouse hepatocytes and differentiated 3T3-L1 adipocytes. Specifically, LPS-induced JNK p46 and NF-κB p65 phosphorylation states and IL-1β and TNFα mRNAs in cGAMP-treated WT primary mouse hepatocytes were significantly lower than their respective levels in control-treated hepatocytes (Fig. 5C,D). In 3T3-L1 adipocytes, the anti-inflammatory effect of cGAMP was even more pronounced. In particular, LPS-induced JNK p46 phosphorylation states in cGAMP-treated adipocytes were markedly lower than in control-treated adipocytes, and were comparable with JNK p46 phosphorylation states in cGAMP-treated adipocytes in the absence of LPS induction (Fig. 5E). Consistently, LPS-induced IL-1β and TNFα mRNAs in cGAMP-treated adipocytes were significantly lower than their respectively levels in control-treated adipocytes (Fig. 5F).

It is unknown whether exogenous cGAMP activates STING; although endogenous cGAMP activates STING. Considering that STING activation stimulates macrophage expression of inflammatory cytokines^27^, we examined whether exogenous cGAMP and STING activation share similarities in altering LPS-stimulated proinflammatory responses. Accordingly, we treated macrophages, hepatocytes, and/or adipocytes with DMXAA. Compared with control BMDM, DMXAA-treated BMDM displayed significant increases in LPS-induced phosphorylation states of JNK p46 and NFκB p65 (Fig. 5G). Similarly, in both WT hepatocytes and differentiated 3T3-L adipocytes, DMXAA treatment significantly enhanced the effect of LPS on increasing the phosphorylation states of JNK p46 and/or NFκB p65 (Fig. 5G). These results, together with the results from cGAMP-treated cells, suggest that exogenous cGAMP acts distinctly from DMXAA on regulating proinflammatory responses; although the two displayed similar effects on stimulating IFNβ production.

### Exogenous cGAMP suppresses hepatocyte gluconeogenic events and fat deposition through pathway(s) involving Akt

**Figure 6:**
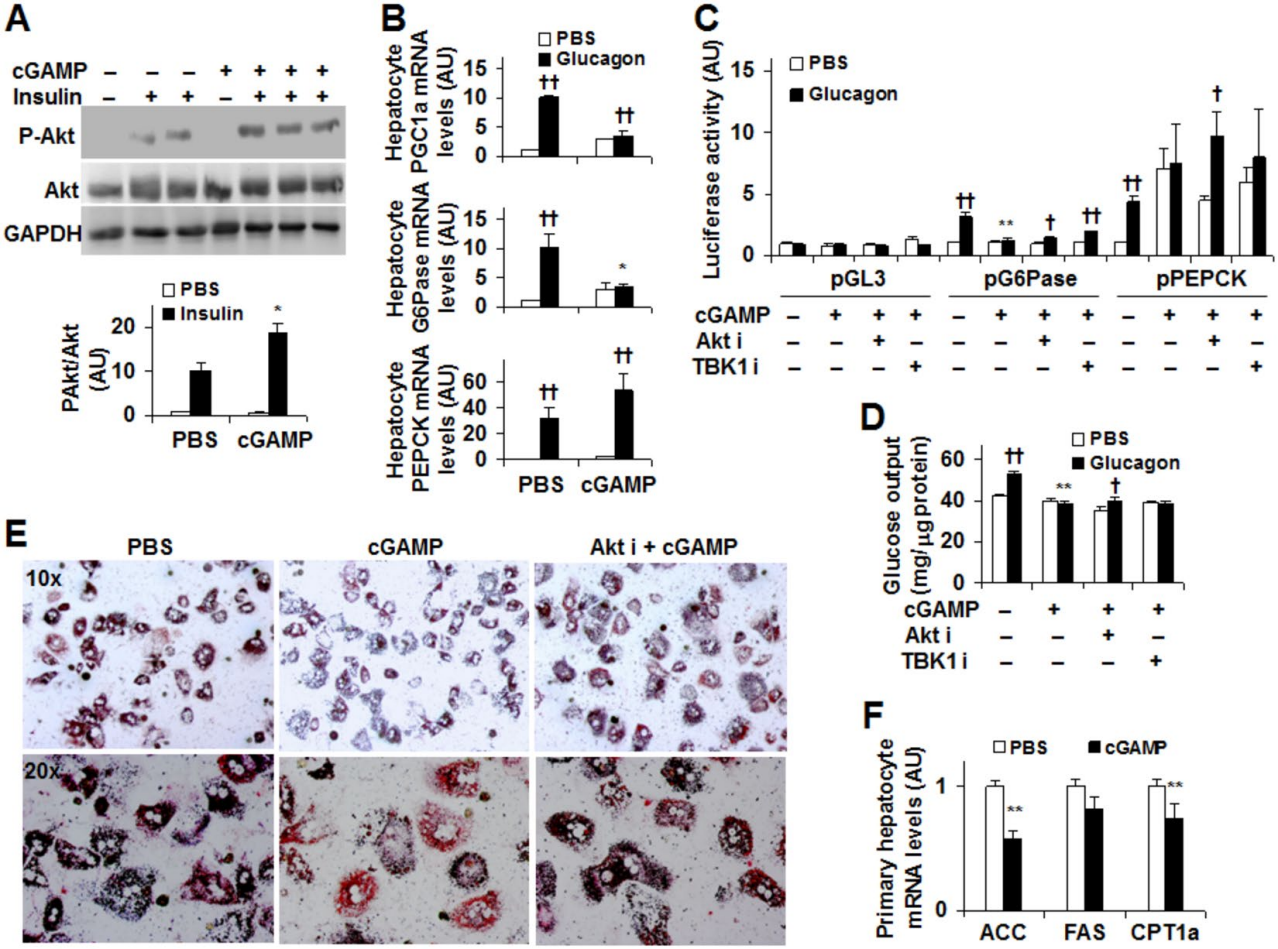
Exogenous cGAMP suppresses hepatocyte gluconeogenic events and fat deposition through pathway(s) involving Akt. (**A**) Hepatocyte insulin signaling. Cropped blots were displayed. Also, full-length blots were included in Supplemental Information (Figure S18). (**B**) Hepatocyte expression of genes for gluconeogenesis. (**C**) Transcription activity of G6Pase promoter and PEPCK promoter. (**D**) Hepatocyte glucose output (HGO). (**E**) Hepatocyte fat deposition. (**F**) Hepatocyte expression of genes related to fat metabolism. For A and B, primary hepatocytes were treated with exogenous cGAMP (20 µg/ml) or PBS for 24 hr in the presence or absence of insulin (100 nM) for the last 30 min (A) or in the presence or absence of glucagon (100 nM) for last 4 hr (B). Insulin signaling was examined using Western blot analysis. Gene expression was examined using real-time PCR. For C, primary hepatocytes were transfected and treated as detailed in Methods. For C–E, primary hepatocytes were treated with cGAMP (20 µg/ml) or PBS for 24 hr in the presence or absence of an Akt inhibitor (MK-2206, 1 µM) or a TBK1 inhibitor (BX795, 1 µM). HGO was measured as described in Methods. For E, to analyze fat deposition, the cells were also treated with or without palmitate (250 µM) for 24 hr. At 1 hr prior to harvest, the cells were stained with Oil-Red-O. For F, primary mouse hepatocytes were treated with cGAMP (20 µg/ml) or PBS for 24 hr. Hepatocyte mRNA levels were analyzed using real-time PCR. For bar graphs, data are means ± S.E. n = 4–8. AU, arbitrary unit. *P < 0.05 and **P < 0.01 cGAMP vs. PBS under the same condition (Insulin-treated condition in A; Glucagon-treated conditions in B; Glucagon-treated conditions in the absence of Akt or TBK inhibition in C and D) for the same gene (in F); and ^†^ *P* < 0.05 and ^††^ *P* < 0.01 Glucagon vs. PBS with the same treatment (PBS or cGAMP) for the same gene (in B) or glucagon-treated condition vs. PBS (in the absence of any other treatment or in the presence of cGAMP with Akt i or TBK1 i in C and D) for the same construct (in C).

Our *in vivo* results from DIO mice suggest a role for cGAMP in associating decreased liver glucose production with improved liver insulin signaling. To address this, we examined the direct effects of exogenous cGAMP on insulin-stimulated Akt phosphorylation. In WT primary hepatocytes, cGAMP treatment increased insulin-induced Akt phosphorylation (Ser473) compared with PBS-treated control (without cGAMP) (Fig. 6A). Because gluconeogenesis critically contributes to HGO, we examined the direct effects of exogenous cGAMP on hepatocyte expression of key genes related to gluconeogenesis. Compared with control, cGAMP treatment blunted the effect of glucagon on increasing hepatocyte G6Pase mRNAs (Fig. 6B) while revealing no significant effects on both the basal and glucagon-stimulated mRNA levels of PPARγ coactivator 1-alpha (PGC1a, a transcriptional coactivator that favors gluconeogenic gene expression) and PEPCK. Next, we performed the reporter assays. As expected, glucagon increased transcription activities of G6Pase promoter and PEPCK promoter in primary mouse hepatocytes compared with the control (in the absence of glucagon) (Fig. 6C). This effect of glucagon, however, was blunted upon treatment with cGAMP; although cGAMP increased basal transcription activities of PEPCK promoter (in the absence of glucagon). We then examined the direct effect of cGAMP on HGO. In the treated hepatocytes, glucagon stimulated HGO and this stimulatory effect was blunted by treatment with cGAMP (Fig. 6D, in the absence or presence of cGAMP alone). Therefore, cGAMP appeared to suppress glucagon-stimulated HGO through decreasing gluconeogenesis.

TBK1 phosphorylates and activates Akt^39^. Thus, we examined whether inhibiting TBK1 and/or Akt alters the effect of cGAMP on hepatocyte gluconeogenic events. In the treated hepatocytes, Akt inhibition significantly weakened or even abolished the effect of cGAMP on suppressing glucagon-induced increase in transcription activities of G6Pase promoter and PEPCK promoter whereas TBK1 inhibition only weakened the effect of cGAMP on suppressing glucagon-induced increase in transcription activity of G6Pase promoter (Fig. 6C). Furthermore, Akt inhibition, but not TBK1 inhibition, significantly weakened the effect of cGAMP on suppressing glucagon-induced increases in HGO (Fig. 6D). These results suggest that cGAMP suppresses glucagon-stimulated gluconeogenic enzyme expression and HGO through pathways involving Akt.

Exogenous cGAMP ameliorated diet-induced hepatic steatosis. We examined the direct effect of cGAMP on hepatocyte fat deposition. Compared with control (bovine serum albumin (BSA)), treatment with palmitate (250 µM, conjugated in BSA) for 24 hr significantly increased hepatocyte fat deposition (Fig. 6E, left two panels). Upon treatment with cGAMP, palmitate-induced fat deposition was significantly decreased (Fig. 6E, middle two panels). This suppressive effect of cGAMP, however, was abolished by the presence of Akt inhibition (Fig. 6E, right two panels), suggesting that Akt is involved in the direct effect of cGAMP on decreasing fat deposition. Also, cGAMP treatment significantly decreased hepatocyte mRNA levels of ACC and CPT1a (Fig. 6F). The latter may reflect an adaptive decrease in fatty acid oxidation in response to decreased hepatocyte fat deposition. Collectively, cGAMP has a direct effect on inhibiting hepatocyte fat deposition, and this inhibitory effect is mediated by pathway(s) involving in Akt.

### Exogenous cGAMP enhances adipocyte Akt phosphorylation and alters adipocyte gene expression

**Figure 7:**
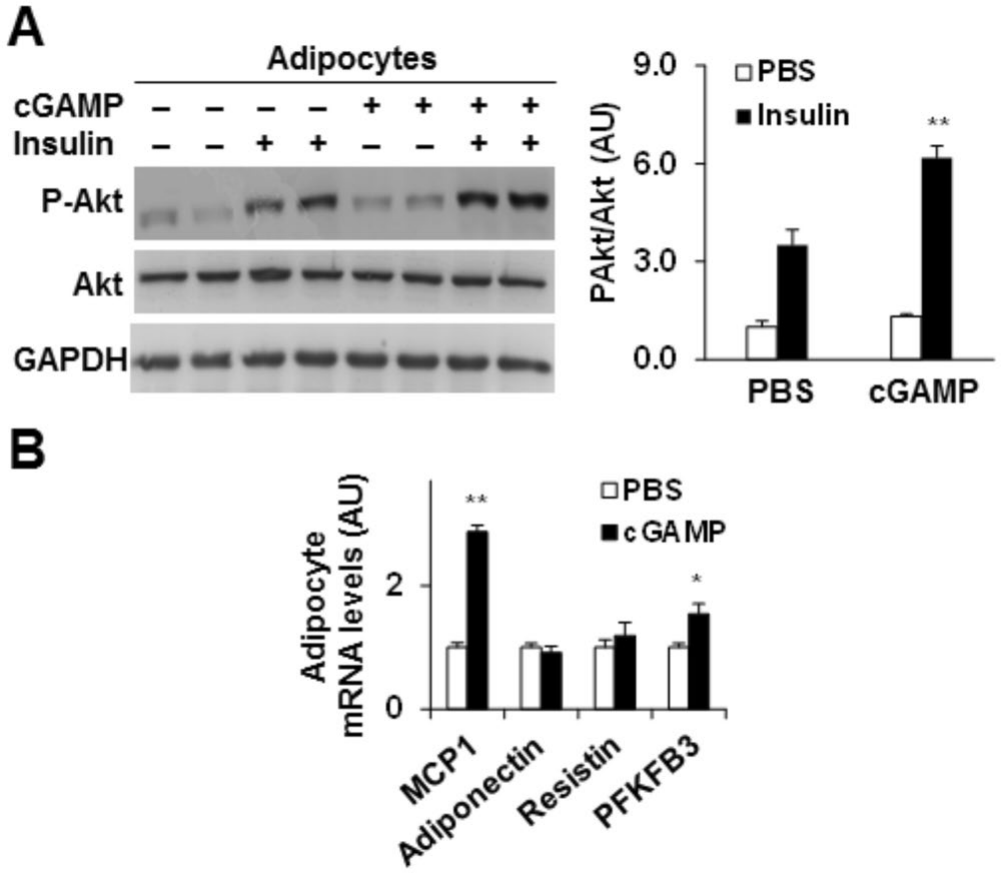
cGAMP improves adipocyte functions. **(A)** Adipocyte insulin signaling. Cropped blots were displayed. Also, full-length blots were included in Supplemental Information (Figure S19). (**B**) Adipocyte gene expression. For A and B, differentiated 3T3-L1 adipocytes were treated with cGAMP (20 mg/ml) or PBS for 24 hr. For A, prior to harvest, adipocytes were treated with or without insulin (100 nM) for the last 30 min. Cell lysates were examined for the phosphorylation and total amount of Akt using Western blot analysis. Blots were quantified using densitometry. For B, the mRNA levels of adipocyte genes were quantified using real-time PCR. For bar graphs, data are means ± S.E. n = 6. *P < 0.05 and **P < 0.01, cGAMP vs. PBS under the same condition (Insulin-stimulated condition in A) for the same gene (in B).

We examined the direct effects of exogenous cGAMP on adipocyte functions. Compared with control, cGAMP treatment significantly enhanced the effect of insulin on increasing Akt phosphorylation states in differentiated adipocytes (Fig. 7A). While it did not significantly alter adipocyte mRNA levels of adiponectin and resistin, cGAMP treatment increased adipocyte mRNA levels of MCP1 and PFKFB3. The latter encodes for the inducible 6-phosphofucto-2-kinase, which protects against adipocyte inflammatory responses and improves adipocyte insulin sensitivity^21, 22^. Taken together, these results suggest that cGAMP has a direct effect on improving adipocyte functions.

## Discussion

Intracellular (endogenous) cGAMP stimulates type I IFN production^13–16, 18^ and is involved in inflammatory responses^19, 20^. We were interested in examining whether and how enzymatically synthesized (exogenous) cGAMP has functions similar to endogenous cGAMP and serves as a modulator of inflammatory responses in the context of regulating metabolic homoeostasis. Using DIO mice, we demonstrated that treatment with exogenous cGAMP suppresses diet-induced inflammatory responses in both the liver and adipose tissue and improves systemic and local metabolic responses. Using cultured macrophages, primary mouse hepatocytes, and adipocytes, we validated for the first time that exogenous cGAMP exerts cell-type-specific anti-inflammatory effects distinctly from STING activation. As such, we provide new insights of how cGAMP links innate immunity and metabolism.

Exogenous cGAMP is functionally active. In support of this, exogenous cGAMP induced macrophage IFNβ production in an STING-dependent manner. This function of exogenous cGAMP is consistent with previous findings that induction of IFNβ production by endogenous cGAMP in macrophages or dendritic cells requires STING^13, 16, 40^. As additional evidence, a single-dose injection of exogenous cGAMP into C57BL/6 J mice induced significant increases in plasma IFNβ levels. This validated the *in vivo* activity of exogenous cGAMP, and led us to examine whether treatment with exogenous cGAMP alters metabolic phenotype of DIO mice. Prior to the current study, there was no published data addressing the role of cGAMP in obesity and related metabolic diseases. Also, there is no effective assay to quantify tissue cGAMP concentrations. We revealed that liver TBK1 phosphorylation was enhanced in DIO mice and positively correlated with diet-induced local (hepatic and adipose tissue) and systemic metabolic dysregulation and insulin resistance. How this occurred is not clear, but may result from STING activation in response to the elevated levels of endogenous cGAMP and/or activation of signaling pathways through TLR4 and RIG-I like receptors^41^. Of significance, treatment with exogenous cGAMP decreased liver TBK1 phosphorylation, which was accompanied with improved local metabolic profile and systemic glucose homeostasis and insulin sensitivity. Because of this, exogenous cGAMP appears to not only function as a negative regulator of TBK1 signaling in the liver, but more importantly, have the capability in altering metabolic homeostasis.

Exogenous cGAMP is also active from the perspective of regulating proinflammatory responses as this is supported by evidence from both *in vivo* and *in vitro* studies. In DIO mice, treatment with exogenous cGAMP significantly ameliorated diet-induced inflammatory responses in both the liver and adipose tissue. In cultured cells, the effects of exogenous cGAMP on altering the proinflammatory responses were even more interestingly. Notably, treatment of macrophages (BMDM from C57BL/6 J mice) with exogenous cGAMP caused significant increases in LPS-induced phosphorylation of JNK p46 and NFκB p65 and in both basal and LPS-induced IL-1β, IL-6, and/or TNFα mRNAs. These effects of exogenous cGAMP are in agreement with the proinflammatory effects of endogenous cGAMP^17^. However, in both hepatocytes and adipocytes, exogenous cGAMP displayed limited effect on stimulating IFNβ production, but exerted anti-inflammatory effects, which is opposite to cGAMP actions on enhancing macrophage proinflammatory activation. Given this, our findings serve as the first evidence that exogenous cGAMP is capable of generating cell-type-specific anti-inflammatory effects. If the same also occurred *in vivo*, the effects of exogenous cGAMP on hepatocytes and adipocytes would dominate over the effect of exogenous cGAMP on macrophages, since cGAMP decreased the proinflammatory responses in both the liver and adipose tissue. This appeared to be the case. In particular, treatment with cGAMP inhibited adipose tissue macrophage M1 activation in DIO mice. A likely explanation is that the anti-inflammatory effects of adipocyte factor(s) generated in response to cGAMP treatment reversed or countered the proinflammatory effects of cGAMP on adipose tissue macrophages. Nevertheless, further studies are needed to validate this possibility, and to explore whether the same also occurs in the liver.

It is a significant finding that exogenous cGAMP acts distinctly from STING activation on regulating the proinflammatory responses. Considering that endogenous cGAMP recruits and activates STING, we postulated that exogenous cGAMP also functions through activating STING. This, however, was not the case from the perspective of regulating proinflammatory responses. In cultured cells, STING activation by DMXAA significantly increased the proinflammatory responses in macrophages, hepatocytes, and adipocytes. In contrast, exogenous cGAMP exhibited a moderate anti-inflammatory effect in primary mouse hepatocytes, and a robust anti-inflammatory effect in differentiated adipocytes although enhancing the proinflammatory activation of macrophages. Clearly, the effects of exogenous cGAMP on altering the proinflammatory responses are distinct from those of STING activation. The underlying mechanisms, however, remain to be explored.

It is a novel finding that exogenous cGAMP has direct effects on suppressing HGO and hepatocyte fat deposition and on improving hepatocyte and adipocyte insulin signaling. Of interest, our findings associate Akt with improved metabolic responses. As shown in published studies, endogenous cGAMP promotes the formation of an STING:TBK1 complex, thereby activating downstream signaling events^13, 16, 40^. In addition, TBK1 can phosphorylate and activate Akt^39^. In the present study, we showed that exogenous cGAMP decreased liver TBK1 phosphorylation. Considering this, exogenous cGAMP may activate Akt through pathways other than activating STING and/or TBK1. As supporting evidence, TBK1 inhibition did not significantly alter the suppressive effect of cGAMP on glucagon-stimulated HGO and PEPCK promoter transcription activity in primary hepatocytes. These findings suggest that TBK1 plays a limited role in cGAMP regulation of hepatocyte gluconeogenesis. Unlike TBK1, Akt is needed for cGAMP actions on gluconeogenic events since Akt inhibition weakened the suppressive effect of cGAMP on glucagon-stimulated G6Pase and PEPCK transcription and HGO. Collectively, our findings argue in favor that cGAMP has a novel role in suppression of hepatic gluconeogenesis, and this role is likely mediated by Akt. When investigating Akt involvement in cGAMP regulation of hepatocyte fat deposition, we also demonstrated that Akt inhibition abolished the effect of cGAMP on suppressing palmitate-induced hepatocyte fat deposition. Thus, it is conceivable that cGAMP activation of Akt is key to its metabolic effects. However, it remains to be explored whether cGAMP activation of Akt requires the anti-inflammatory effects of cGAMP.

In summary, the present study reveals that exogenous cGAMP functions as a regulator that links innate immunity and metabolic homeostasis. At the integrative level, cGAMP protects against diet-induced inflammation in both the liver and adipose tissue and ameliorates diet-induced metabolic dysregulation and insulin resistance. At the cellular level, cGAMP exerts cell-type-specific anti-inflammatory effects distinctly from STING activation, and improves hepatocyte and adipocyte metabolic responses. Therefore, the novel findings not only provide a better understanding of how the innate immune system interacts with metabolic system, but also validate the potential applications of cGAMP or cGAMP-based approaches for prevention and treatment of inflammatory and metabolic diseases.

## Electronic supplementary material

Supplementary Information
