# Supplementary material for: Cyclic GMP-AMP Ameliorates Diet-induced Metabolic Dysregulation and Regulates Proinflammatory Responses Distinctly from STING Activation
Source: Sci Rep. 2017 Jul 25;7:6355. doi: 10.1038/s41598-017-05884-y (PMC5526935; doi:10.1038/s41598-017-05884-y)
Supplement: Supplementary file 1 — Supplementary Information [file 41598_2017_5884_MOESM1_ESM.pdf]

Cyclic GMP-AMP Ameliorates Diet-induced Metabolic Dysregulation and Regulates Proinflammatory Responses Distinctly from STING Activation

Xin Guo, Chang Shu, Honggui Li, Ya Pei, Shih-Lung Woo, Juan Zheng, Mengyang Liu, Hang Xu, Rachel Botchlett, Ting Guo, Yuli Cai, Xinsheng Gao, Jing Zhou, Lu Chen, Qifu Li, Xiaoqiu Xiao, Linglin Xie, Ke K. Zhang, Jun-Yuan Ji, Yuqing Huo, Fanyin Meng, Gianfranco Alpini, Pingwei Li, and Chaodong Wu

Supplemental Information

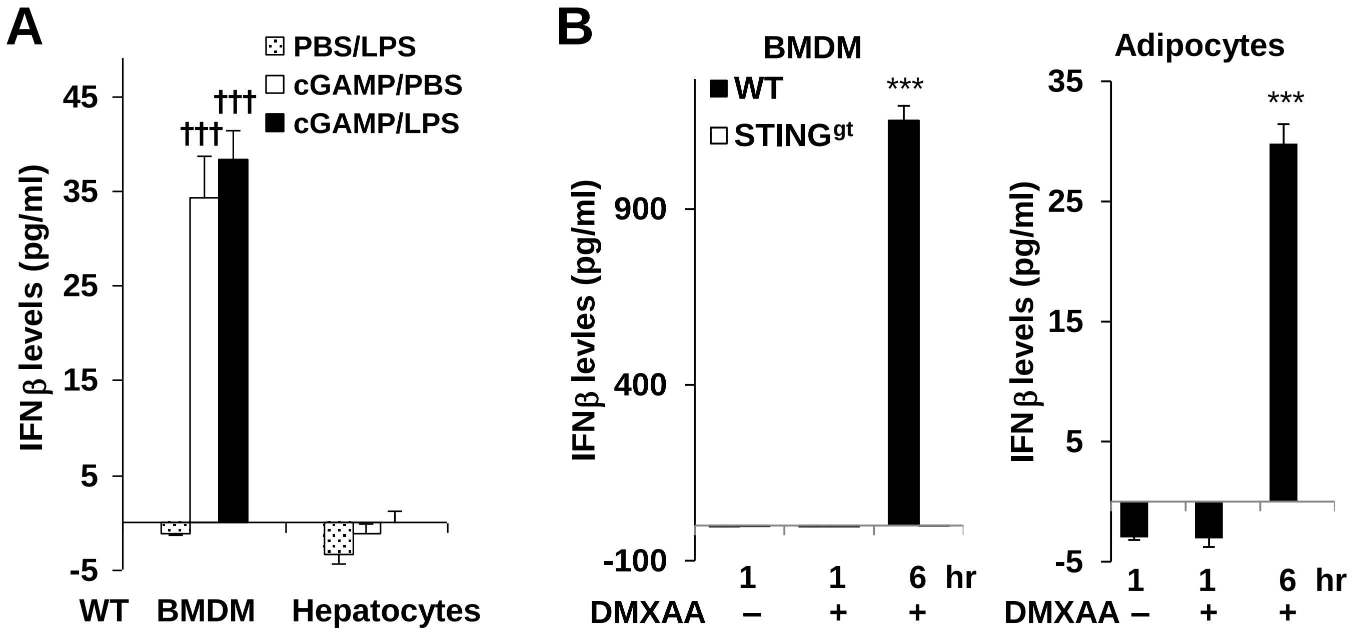

Figure S1. Related to Figure 1: Both cGAMP and DMXAA stimulate IFNβ production in wild-type macrophages, but not STING-disrupted macrophages. (**A**) The effects of lipopolysaccharide (LPS) on interferon beta (IFNβ) production. (**B**) DMXAA stimulation of IFNβ production in macrophages and adipocytes. For A, bone marrow cells were isolated from male wild-type (WT) C57BL/6J mice and STING-disrupted (STING^{gt}) mice and differentiated into macrophages (BMDM). After differentiation, BMDM were treated with PBS or enzymatically synthesized cGAMP (20 µg/ml) for 24 hr in the presence of LPS (20 ng/ml) for the last 6 hr. For B, BMDM were prepared as described in A. Also, adipocytes were differentiated from 3T3-L1 cells. After differentiation, either BMDM or adipocytes were treated with DMXAA (75 µg/ml, 100 × stock in 7.5% $NaHCO_3$) or $NaHCO_3$ solution (Ctrl) for 1 or 6 hr. For A and B, data are means ± S.E. n = 5 – 6. †††, *P* < 0.001 cGAMP/PBS or cGAMP/LPS vs. PBS/LPS for the same cell type; ***, *P* < 0.001 DMXAA at 6 hr vs. Ctrl (in the absence of DMXAA) or DMXAA at 1 hr (for WT BMDM in left bar graph; for adipocytes in right bar graph).

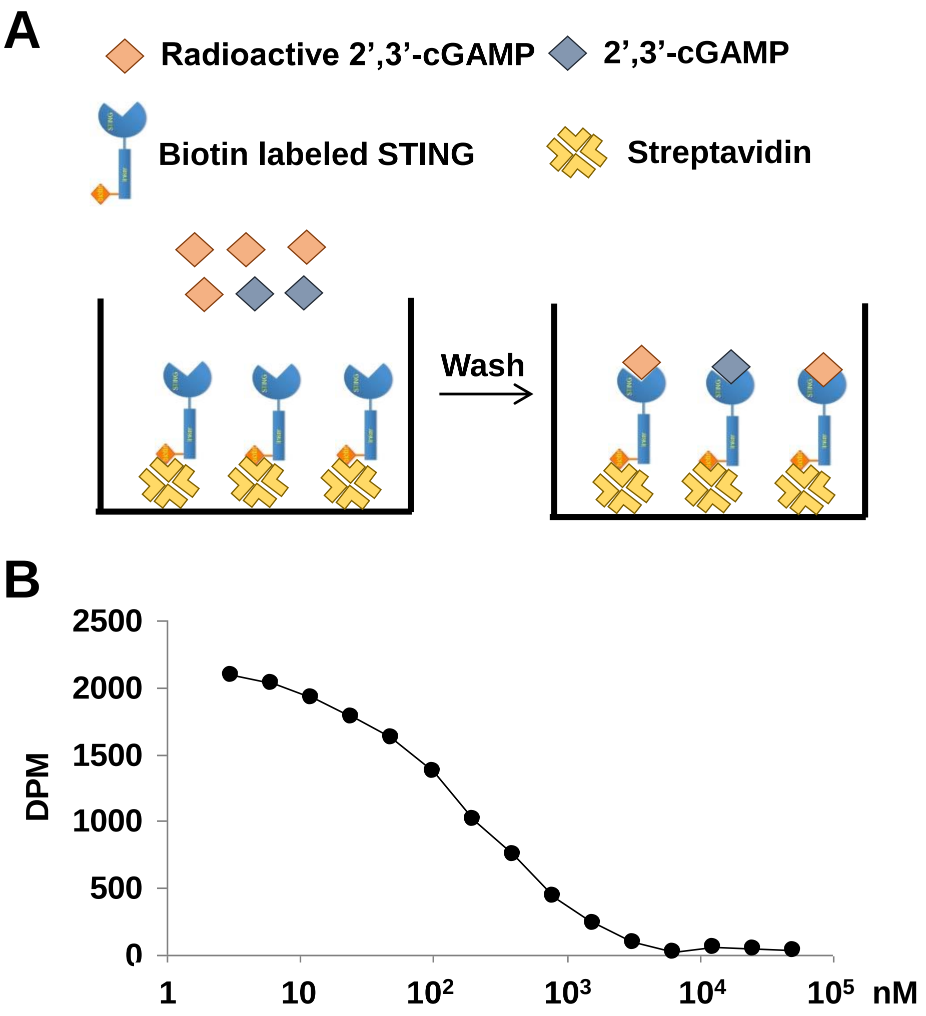

Figure S2. Related to Figure 1: A method for quantification of cGAMP. (**A**) A scheme for competitive cGAMP radioimmunoassay (RIA). (**B**) A cGAMP calibration curve was obtained using the RIA.

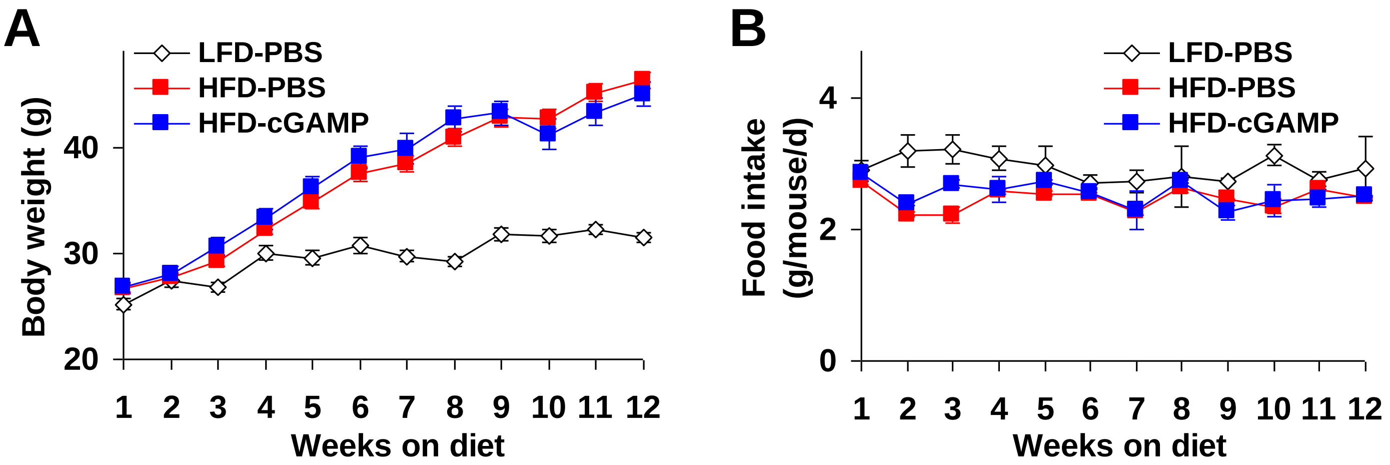

Figure S3. Related to Figure 2: Treatment with cGAMP does not alter body weight and food intake. (**A**) Body weight was recorded weekly during the feeding period. (**B**) Food intake was calculated based on food consumption and expressed as food weight per mouse per day. For A and B, male C57BL/6J mice were fed and treated as described in Figure 2. Data are means ± S.E. n = 10 -12.

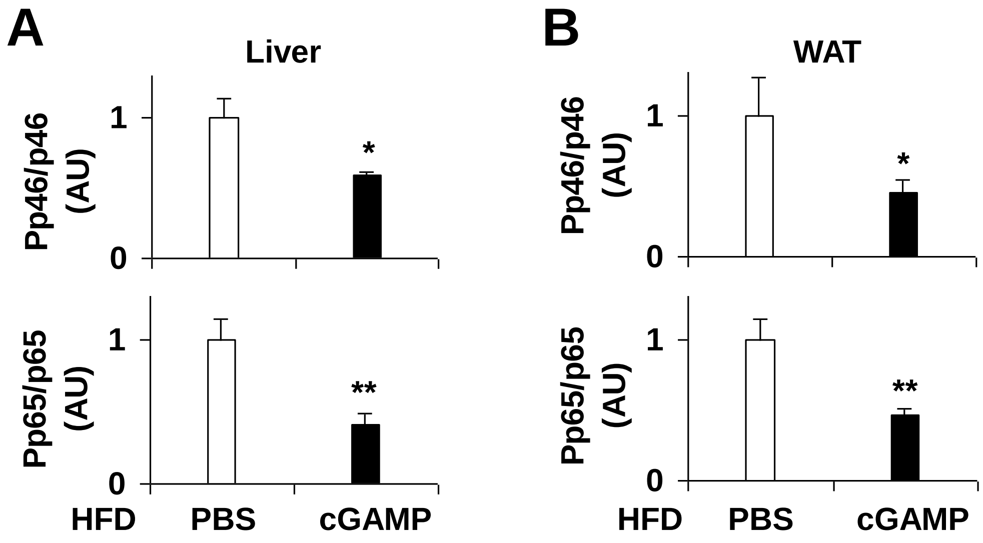

Figure S4. Related to Figure 3: Treatment with cGAMP decreases liver and adipose tissue proinflammatory signaling. (**A**,**B**) Quantitative data of liver (A) and adipose tissue (B) proinflammatory signaling. For A and B, male C57BL/6J mice were fed and treated as described in Figure 3. Liver and adipose tissue proinflammatory signaling was analyzed as described in Figure 3B. The maximum intensity of each band was quantified. Ratios of Pp46/p46 and Pp65/p65 were normalized to GAPDH and adjusted relative to the average of PBS-treated control, which was arbitrarily set as 1 (AU). Data are means ± S.E. n = 5 – 7. *, $P < 0.05$ and **, $P < 0.01$ cGAMP vs. PBS on an HFD.

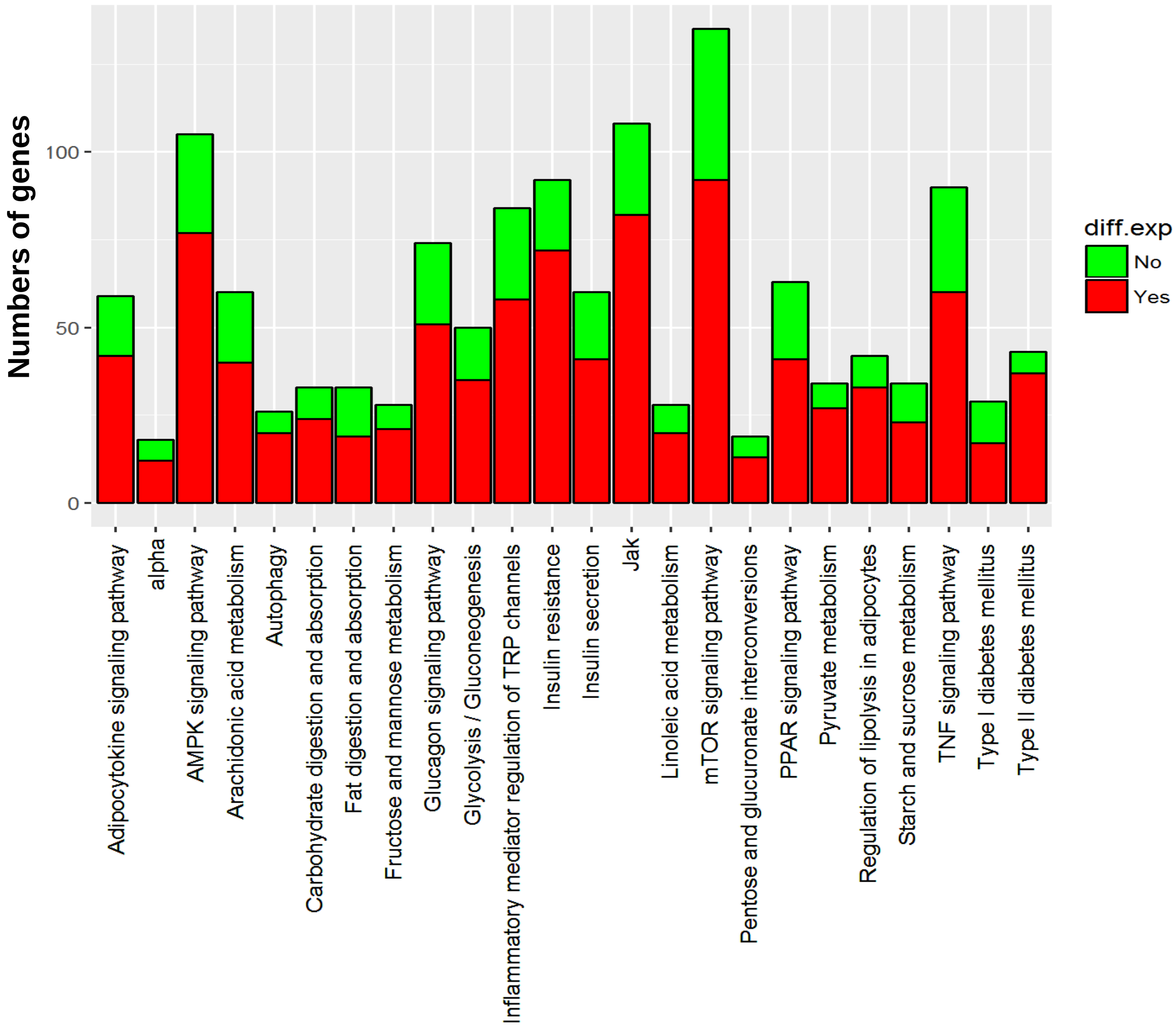

Figure S5. Related to Figure 4: Treatment with cGAMP activates the gene signaling pathways of glucose and fat metabolism. Male C57BL/6J mice were fed and treated as described in Figure 4. Liver RNA samples were subjected to microarray assays. Comparisons were made between HFD-cGAMP mice and HFD-PBS mice for differentially expressed genes. All the 24 selected KEGG pathways were significantly induced or repressed by cGAMP (FDR < 0.001). The bars represent the numbers of differentially expressed genes (red) and non-differentially expressed genes (green). n.genes, the number of genes; diff.exp, differentially expressed.

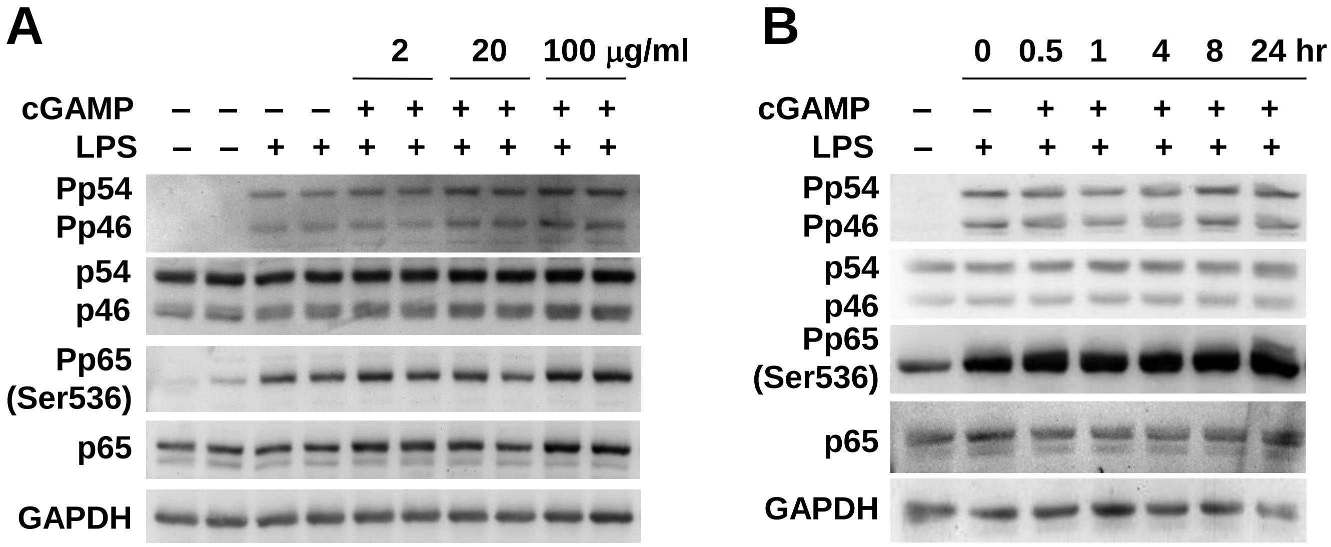

Figure S6. Related to Figure 5: cGAMP enhances the proinflammatory activation of wild-type macrophages. (**A**) Dose-response study of cGAMP effects on macrophage inflammatory signaling. (**B**) Time-course study of cGAMP effects on macrophage inflammatory signaling. For A and B, WT BMDM were examined for proinflammatory signaling using Western blot analyses. For A, BMDM were treated with PBS or cGAMP at the doses indicated for 24 hr in the absence or presence of LPS (100 ng/ml) for the last 30 min. For B, BMDM were treated with PBS or cGAMP (20 μg/ml) for 0.5, 1, 4, 8, and/or 24 hr in the absence or presence of LPS (100 ng/ml) for the last 30 min. (**C**) and (**D**) are full-length blots of A and B, respectively.

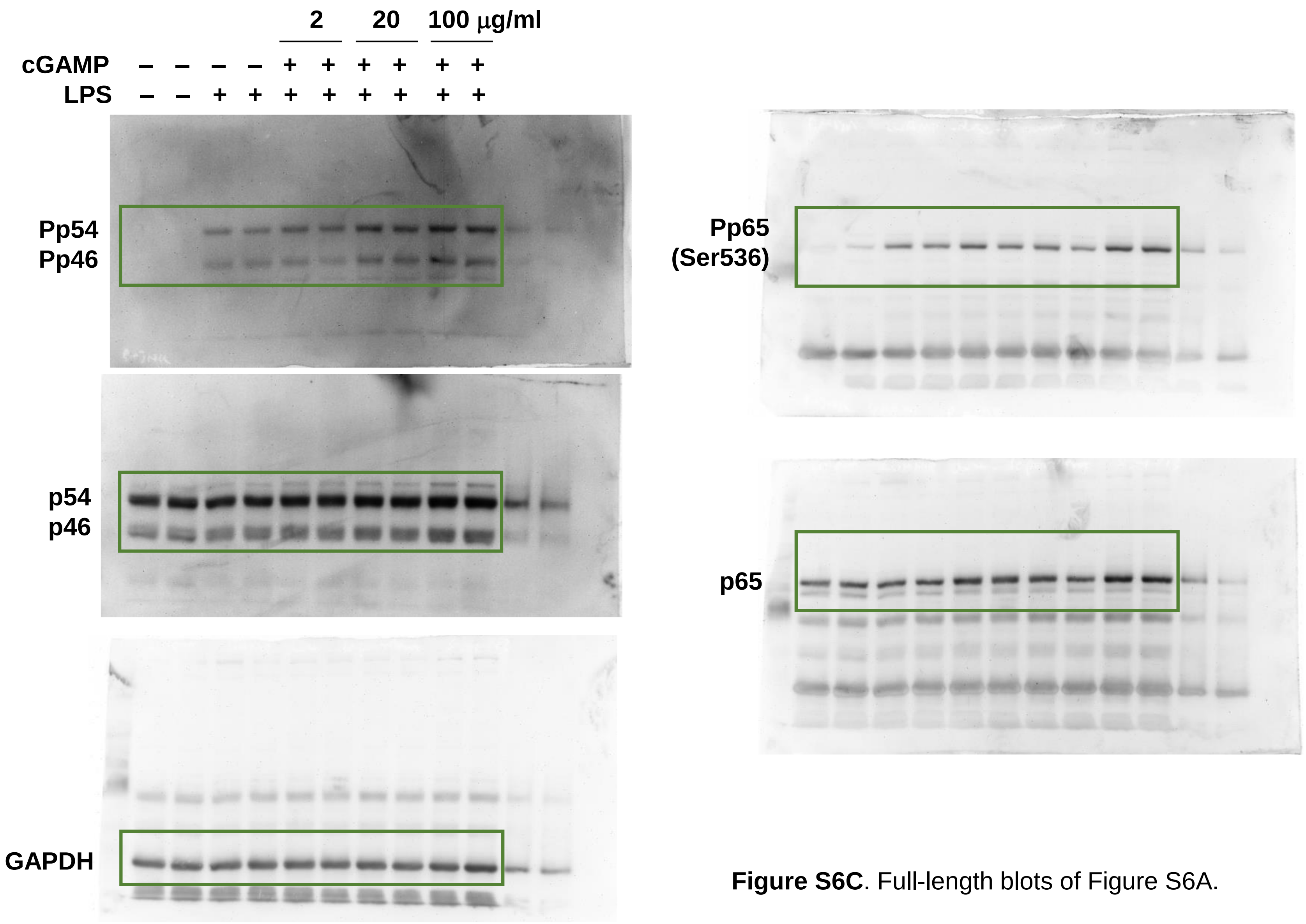

Figure S6C. Full-length blots of Figure S6A.

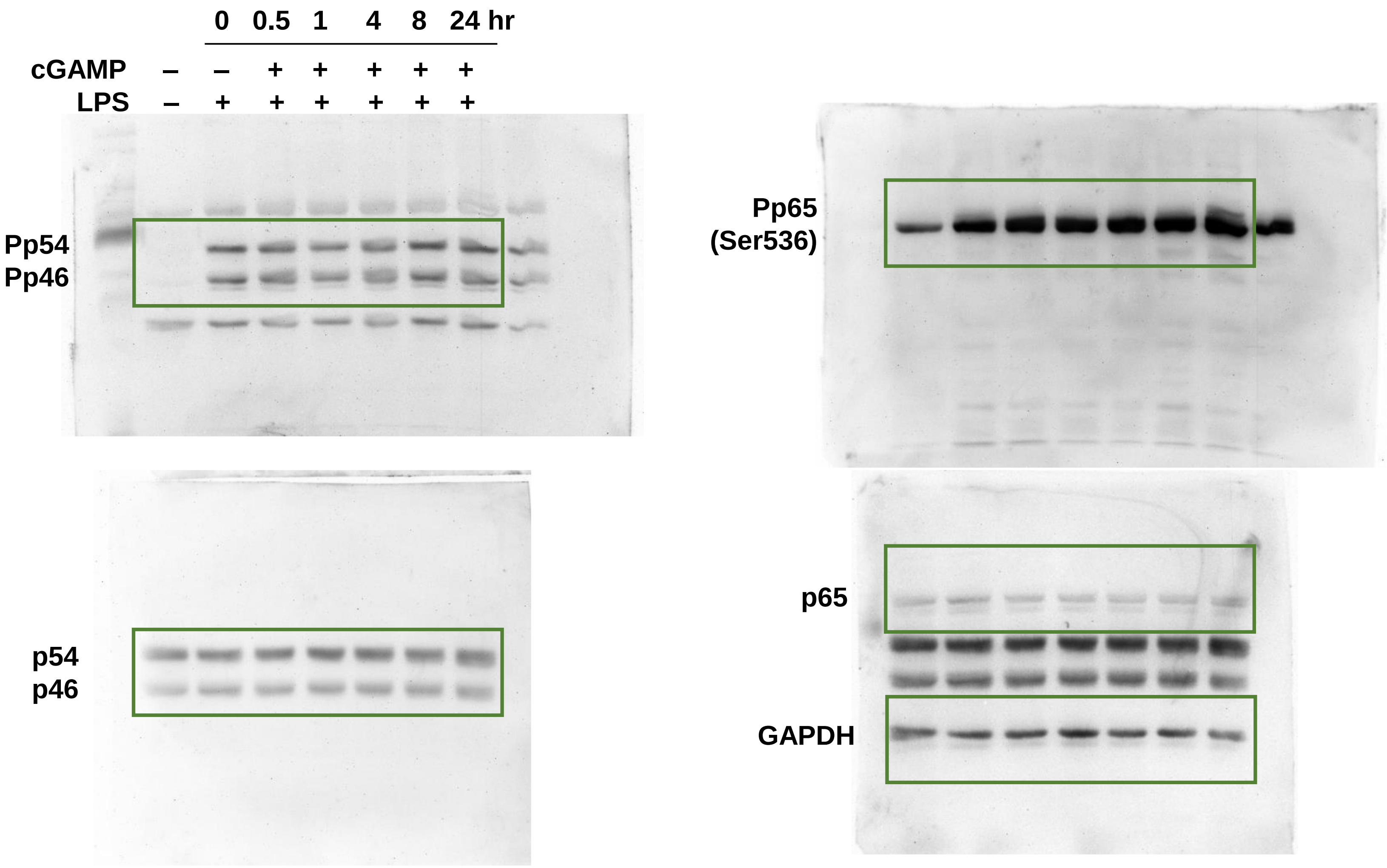

Figure S6D. Full-length blots of Figure S6B.

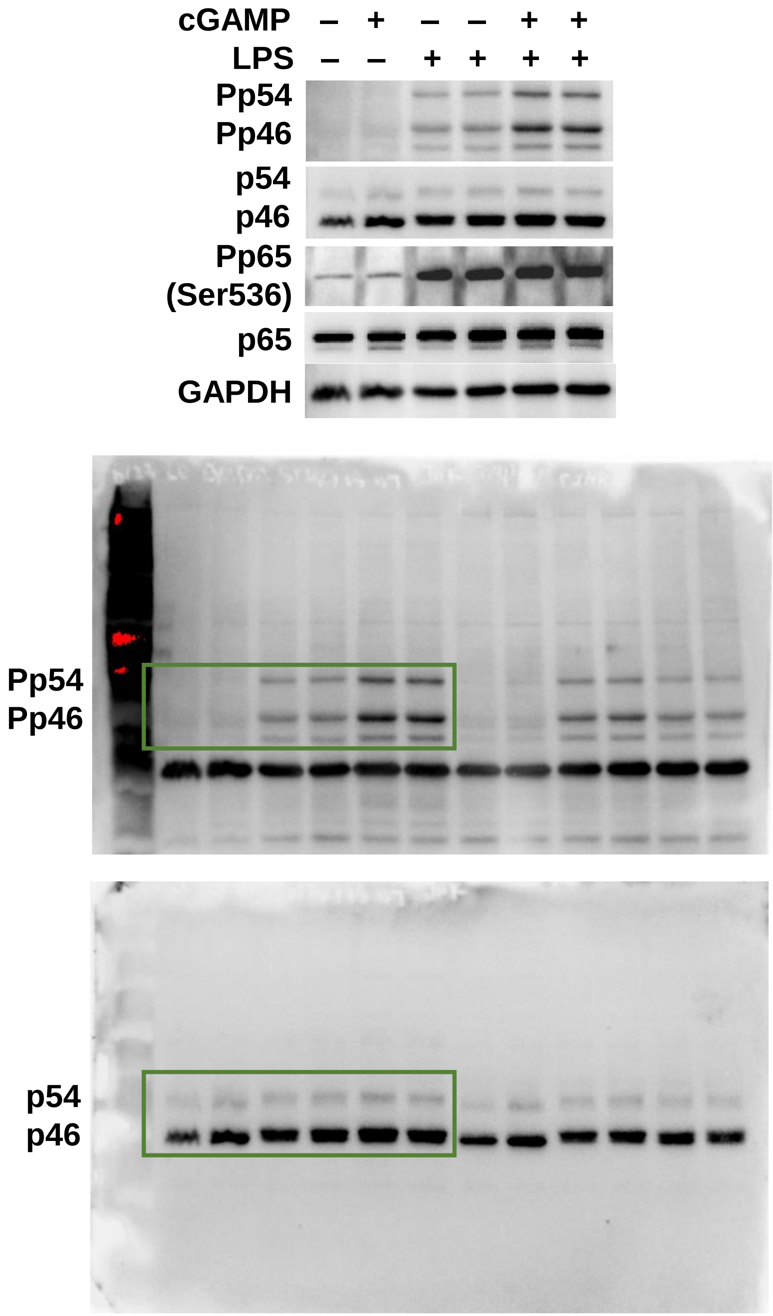

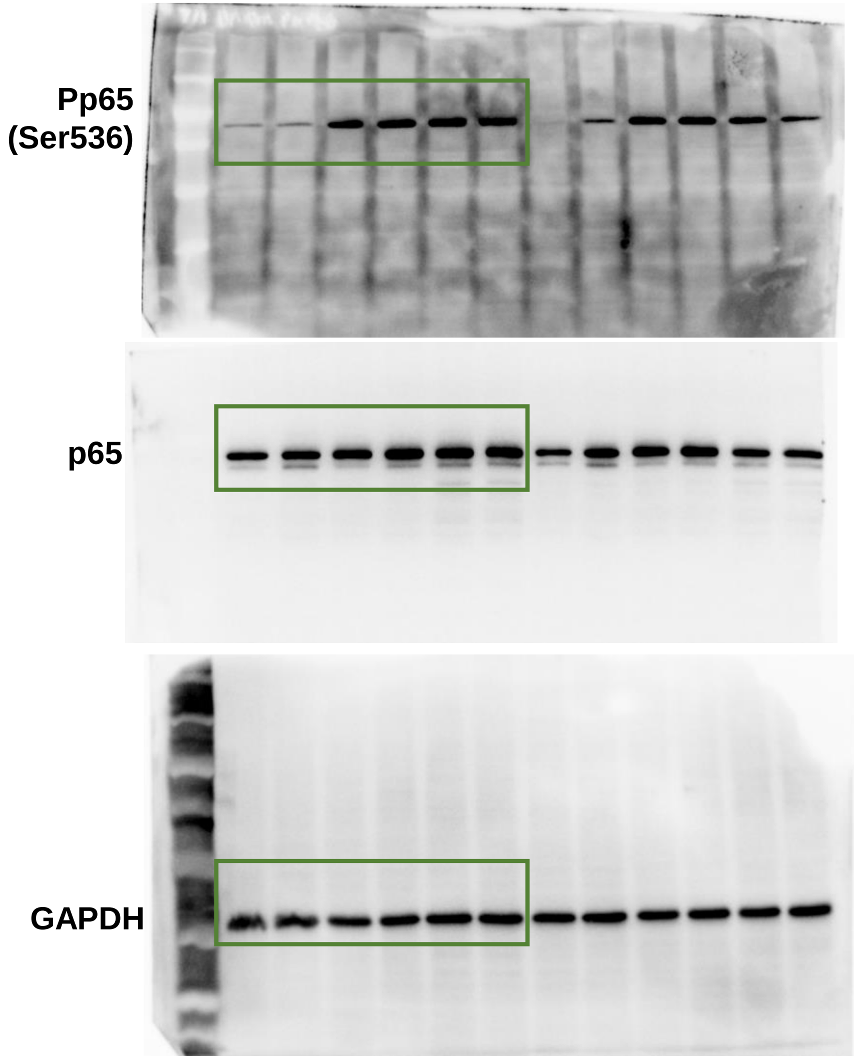

Figure S7. Related to Figure 5: cGAMP enhances the proinflammatory signaling in wild-type macrophages. Bone marrow cells were isolated from wild-type C57BL/6J mice and differentiated into macrophages (BMDM). After differentiation, BMDM were treated with PBS or commercial cGAMP (20 μg/ml; InvivoGen, Cat. Code: tlrl-nacga23-5) for 24 hr in the absence or presence of LPS (100 ng/ml) for the last 30 min. Proinflammatory signaling was examined using Western blot analyses. Top left panels are cropped blots. The rest are full-length blots.

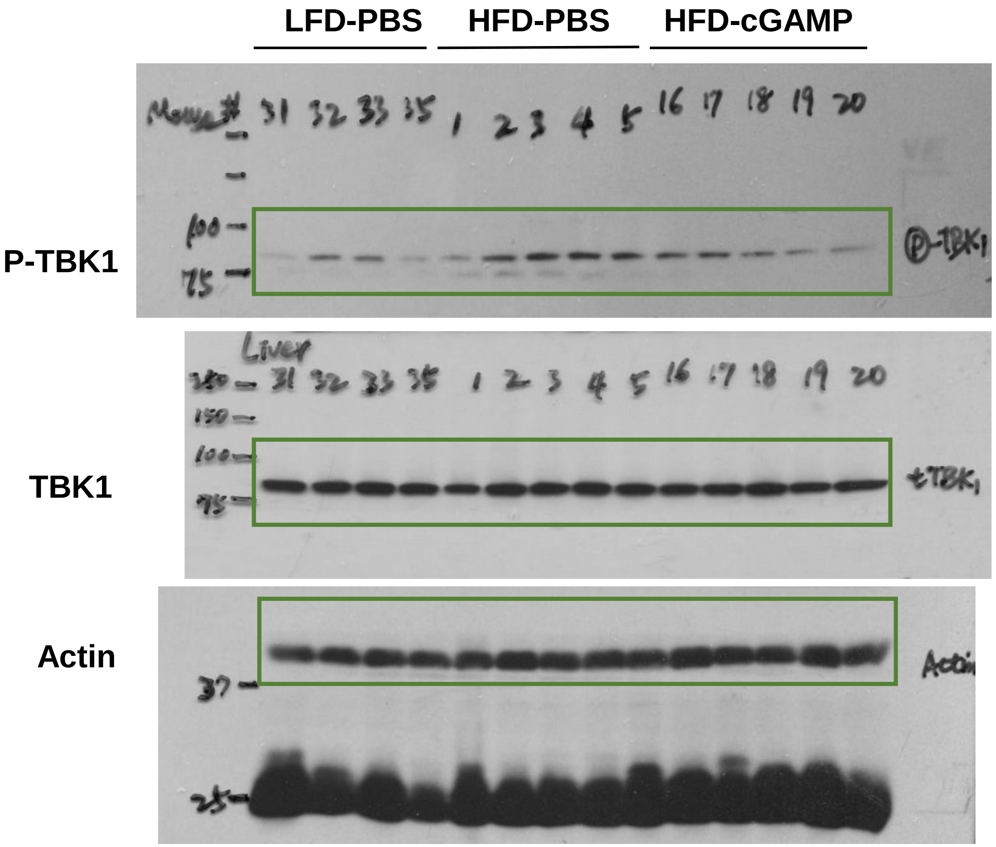

Figure S8. Related to Figure 2: Full-length blots of liver TBK1 phosphorylation.

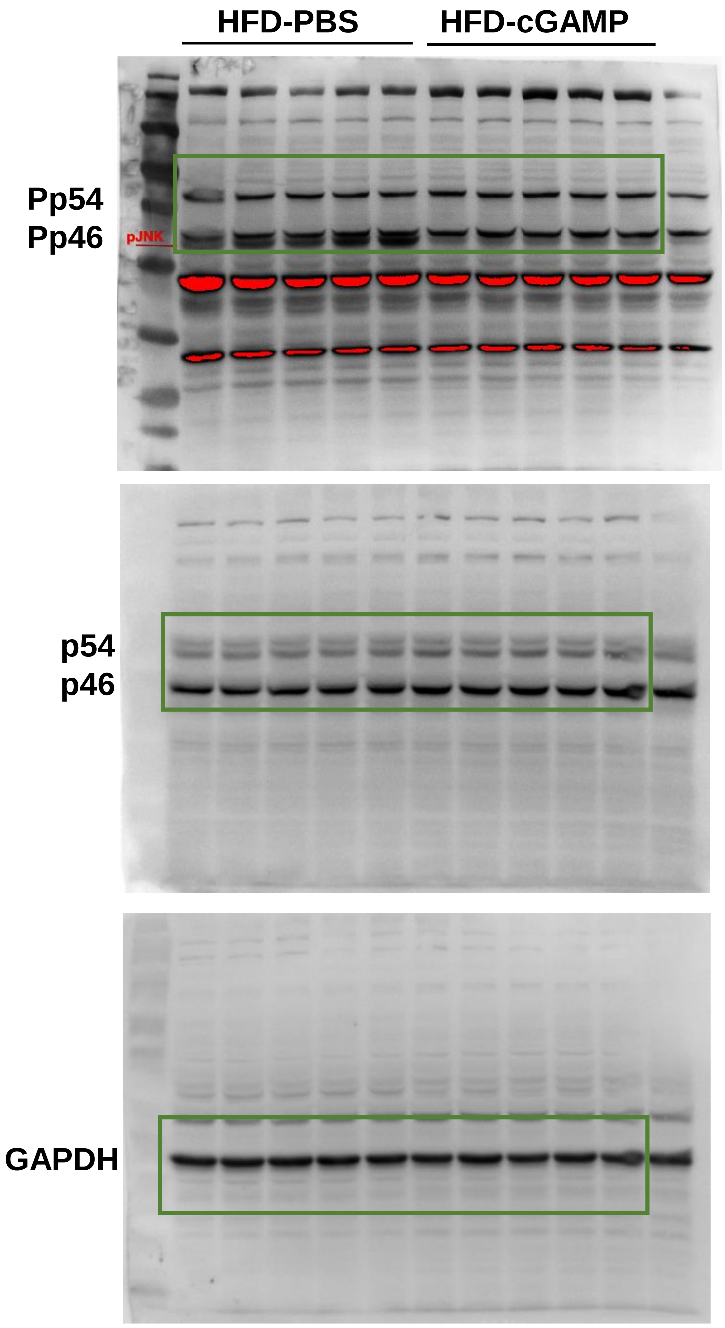

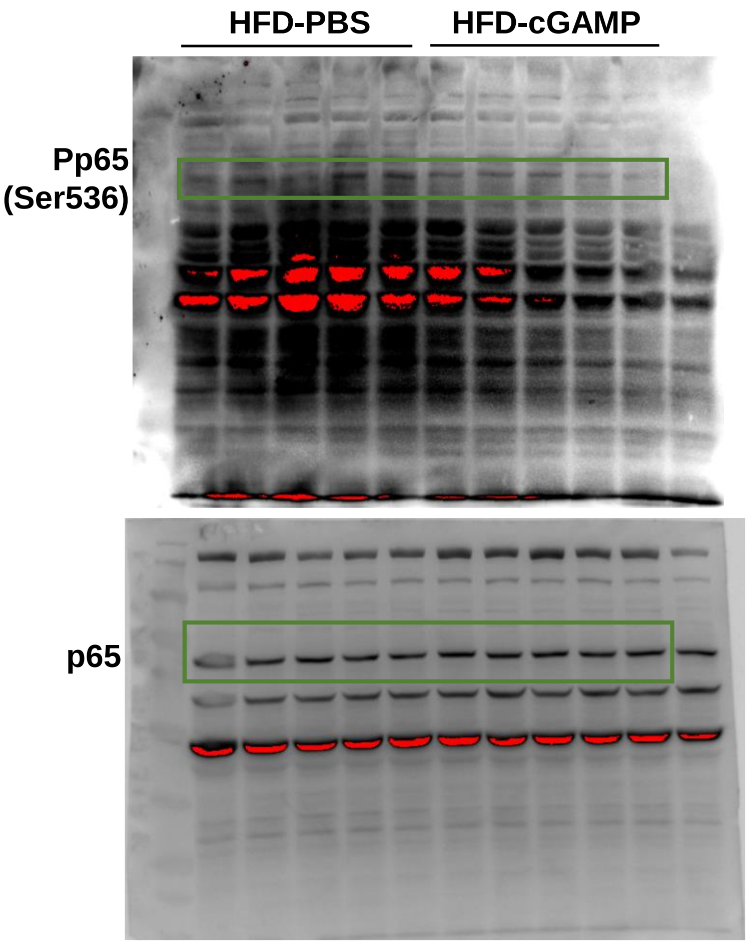

Figure S9. Related to Figure 3: Full-length blots of liver inflammatory signaling.

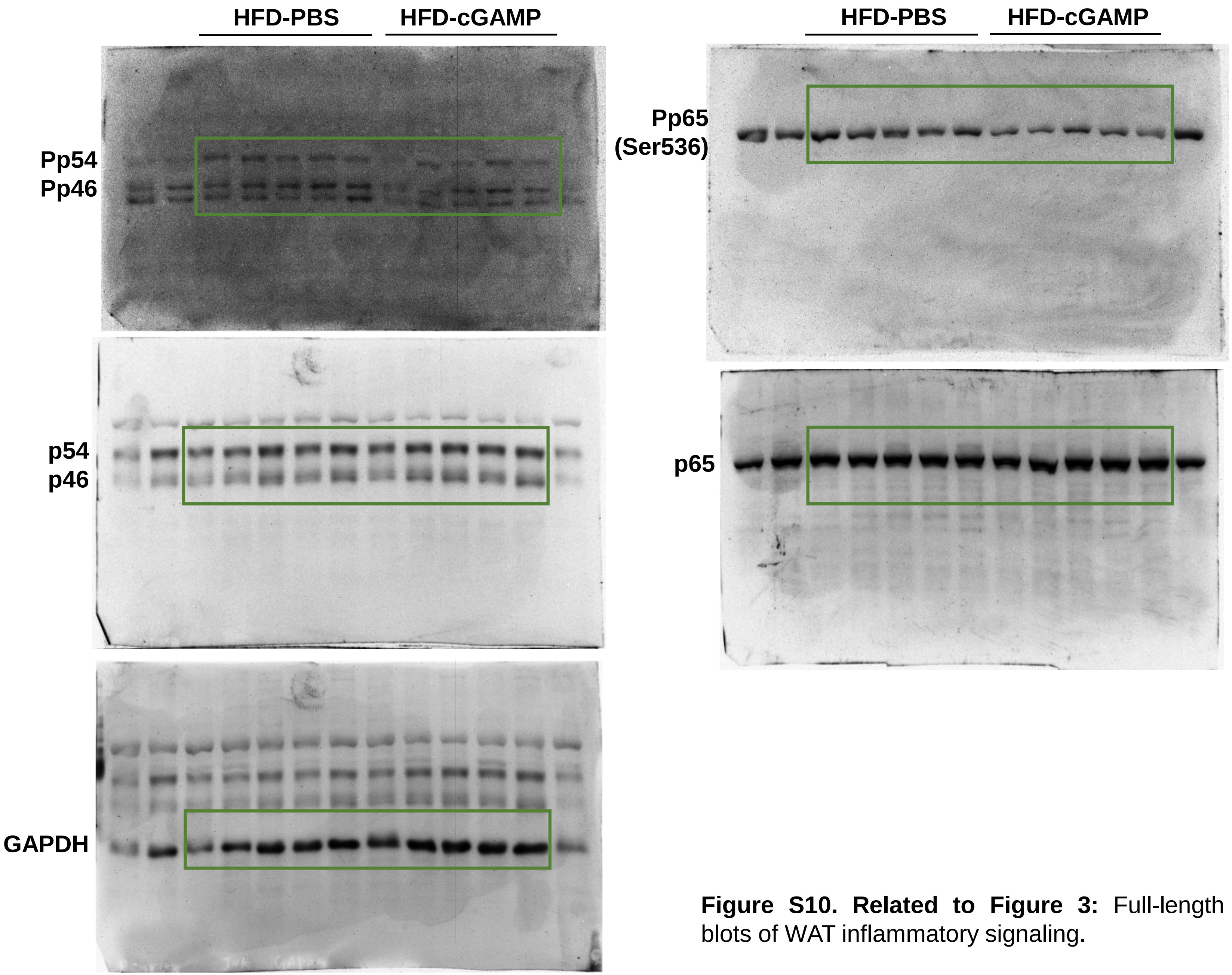

Figure S10. Related to Figure 3: Full-length blots of WAT inflammatory signaling.

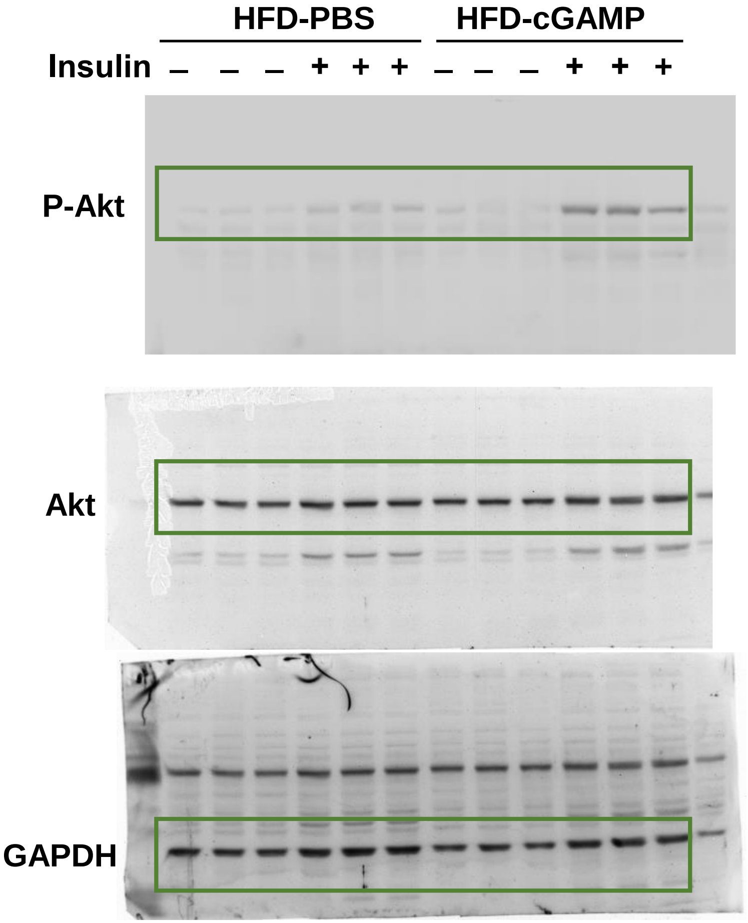

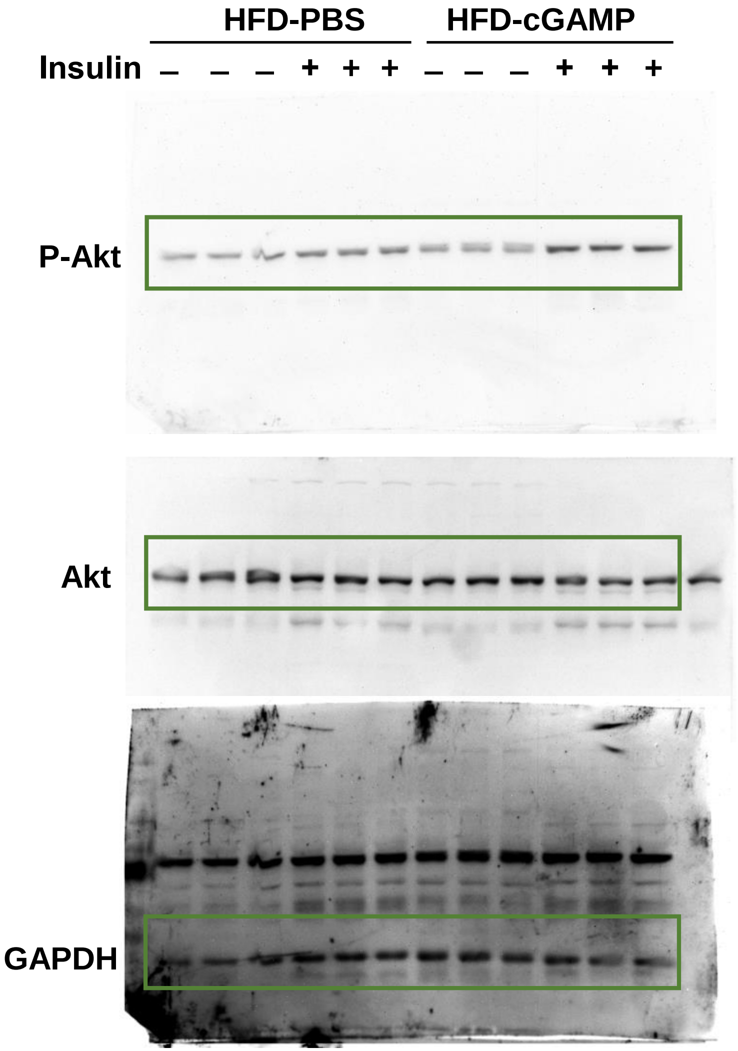

Figure S11. Related to Figure 4: Full-length blots of Akt phosphorylation in livers (left panels) and WAT (right panels).

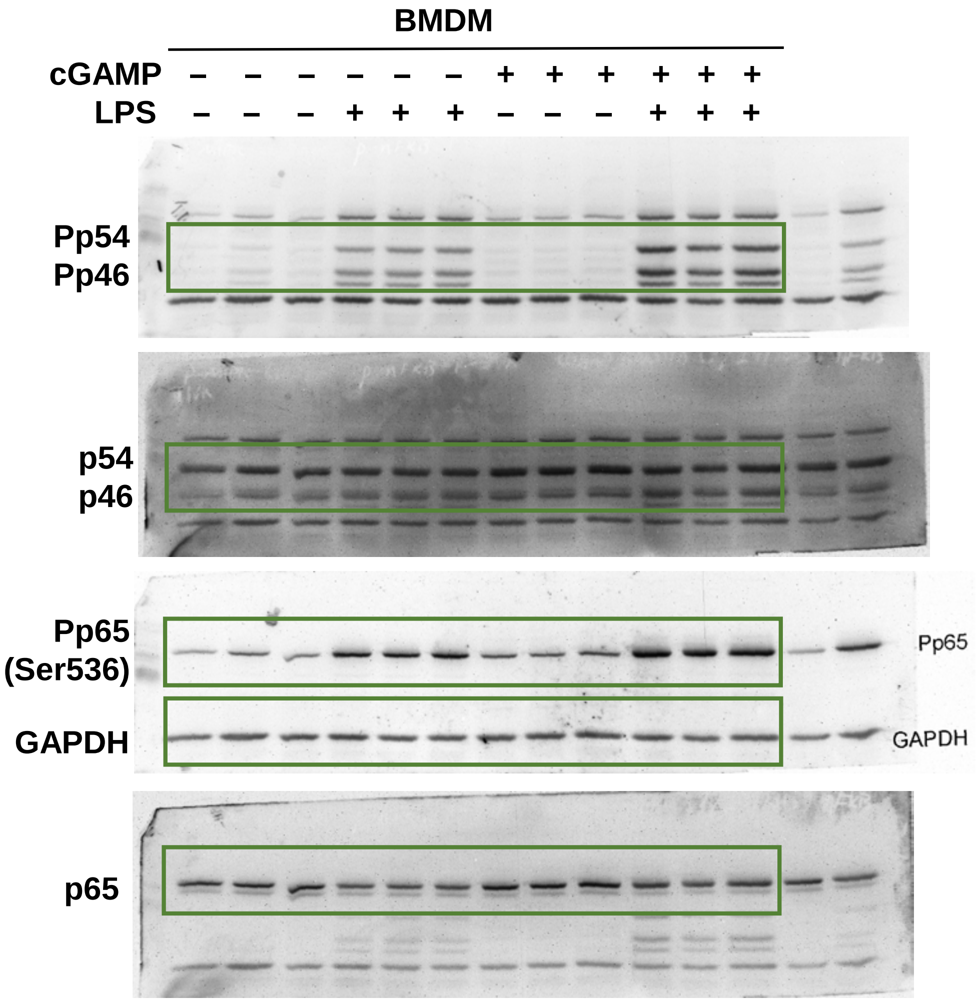

Figure S12. Related to Figure 5A: Full-length blots of BMDM inflammatory signaling.

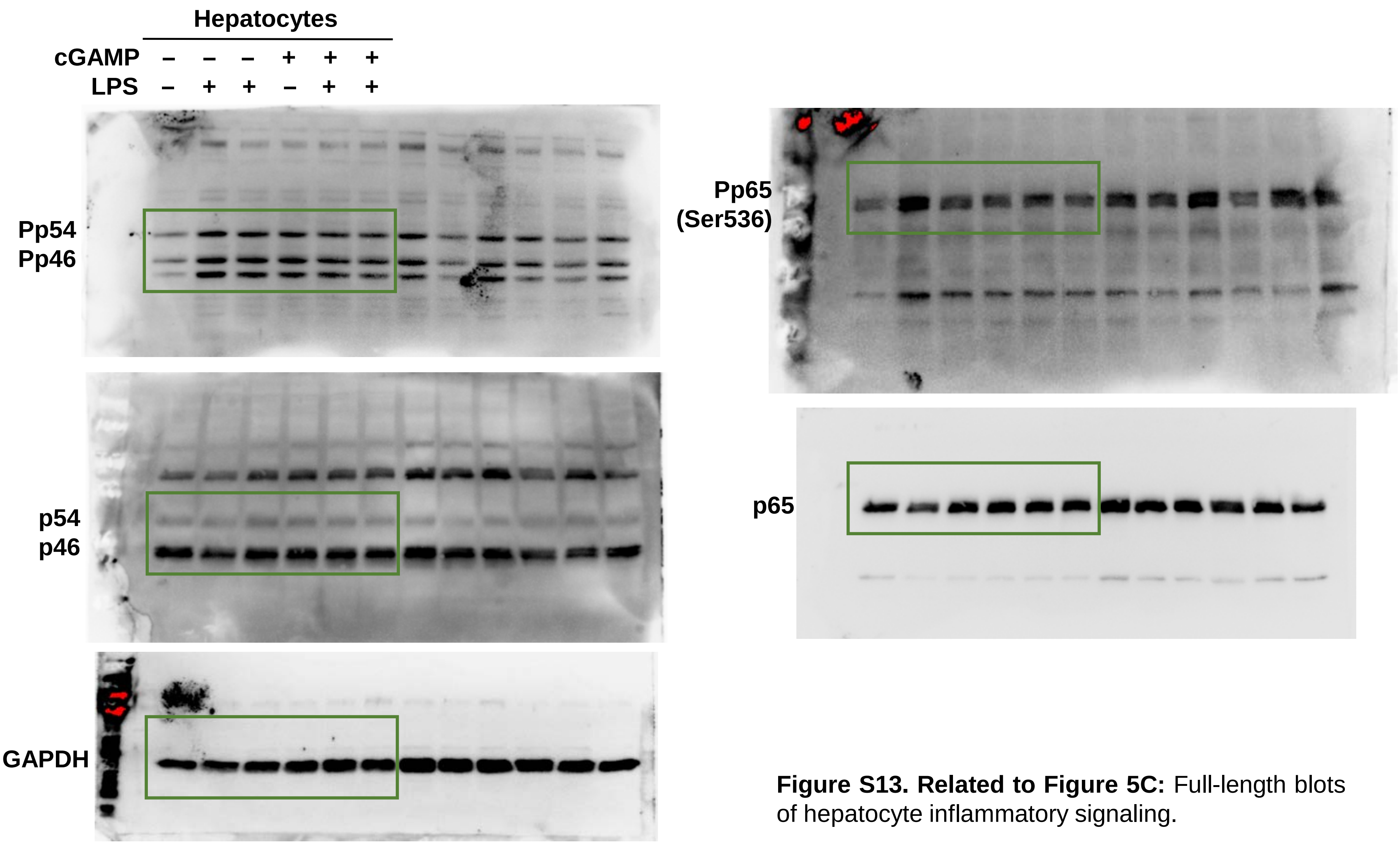

Figure S13. Related to Figure 5C: Full-length blots of hepatocyte inflammatory signaling.

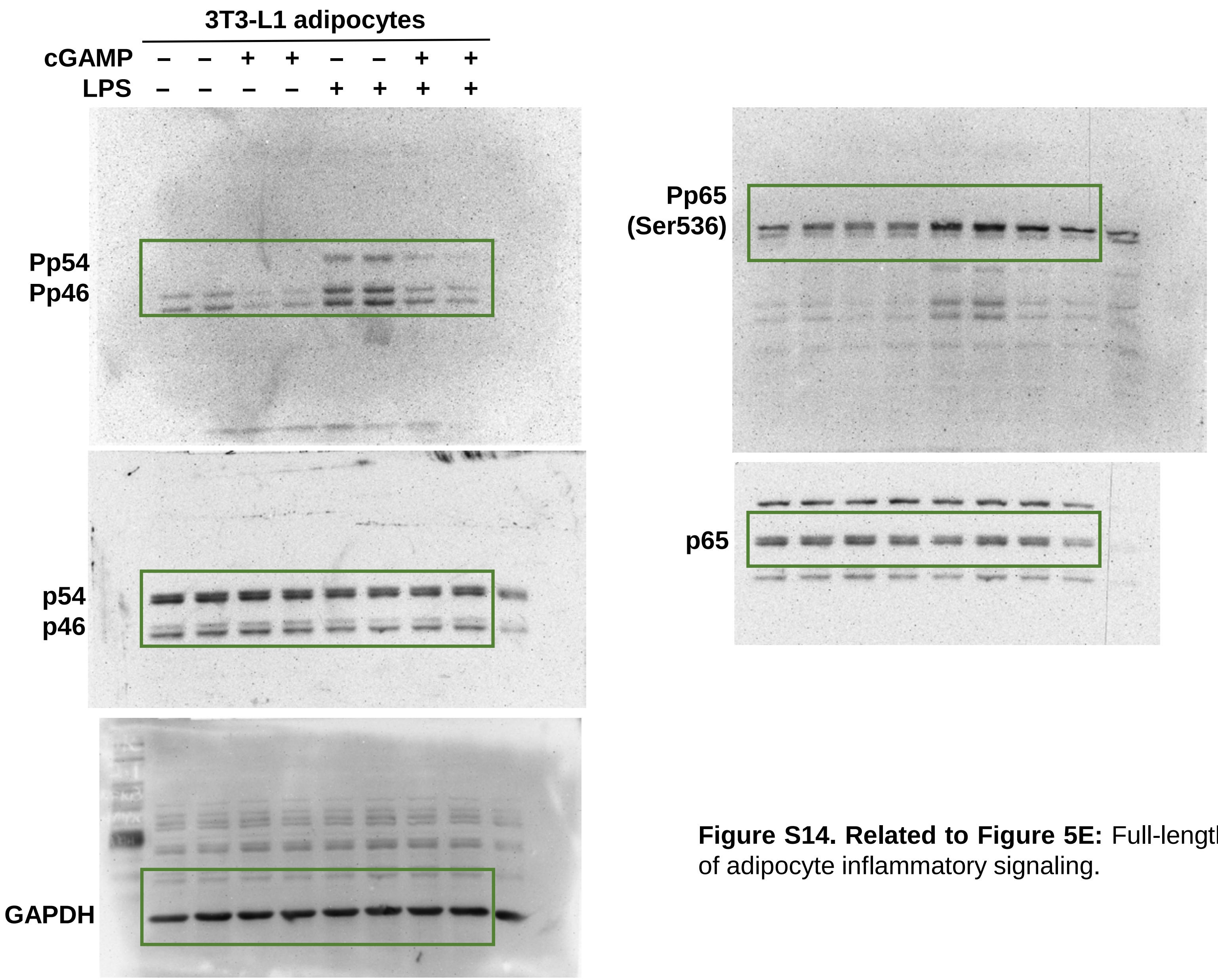

Figure S14. Related to Figure 5E: Full-length blots of adipocyte inflammatory signaling.

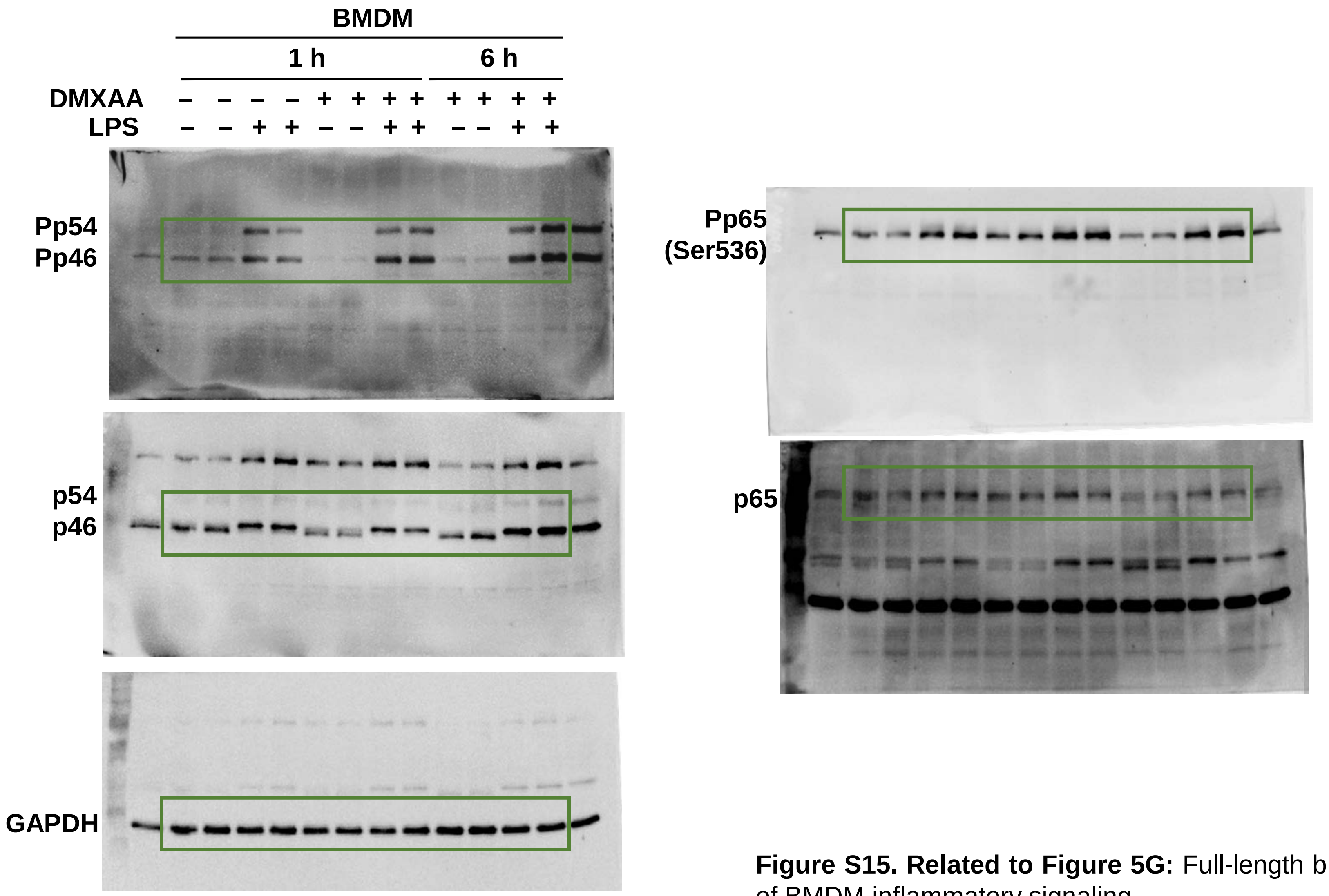

Figure S15. Related to Figure 5G: Full-length blots of BMDM inflammatory signaling.

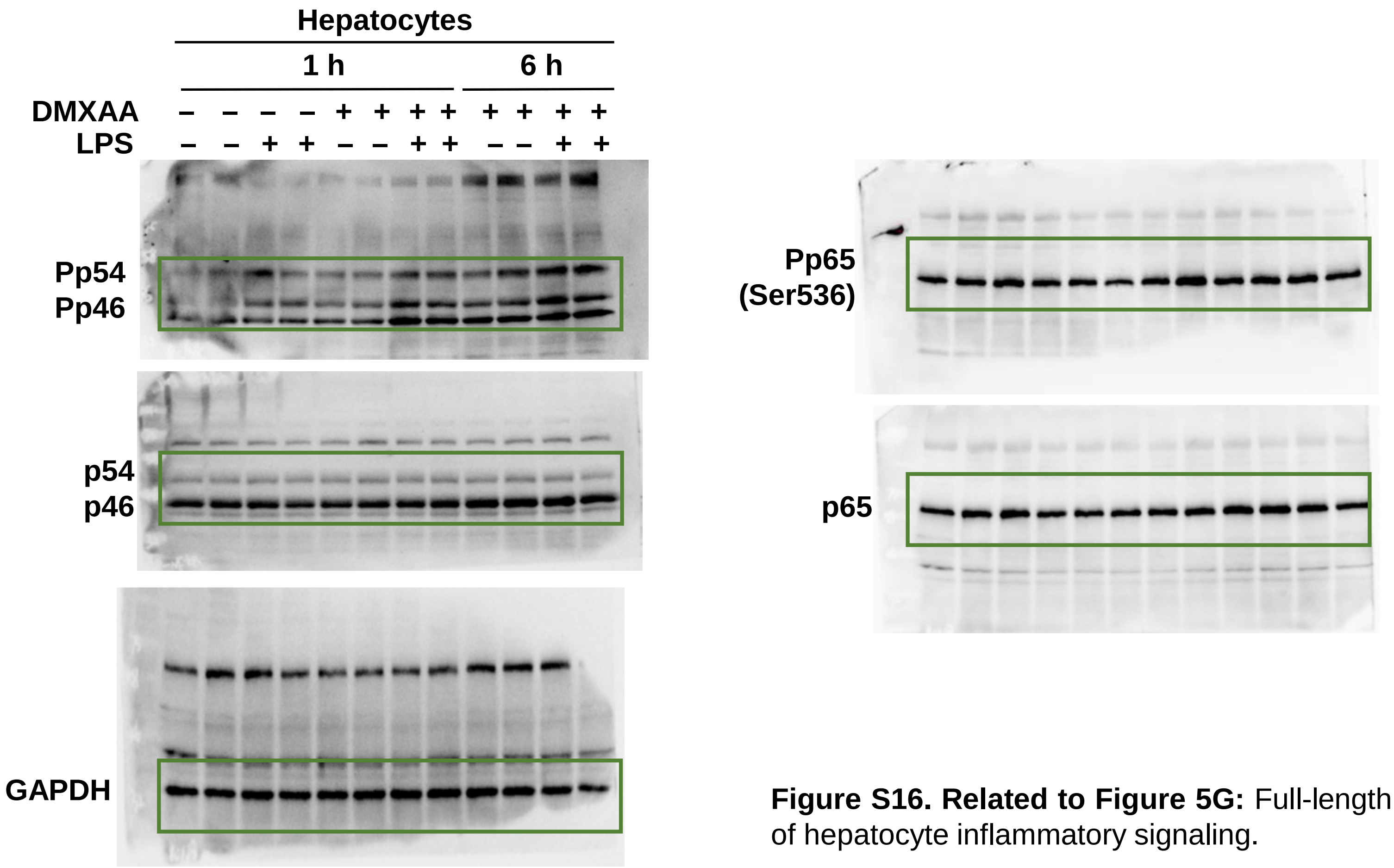

Figure S16. Related to Figure 5G: Full-length blots of hepatocyte inflammatory signaling.

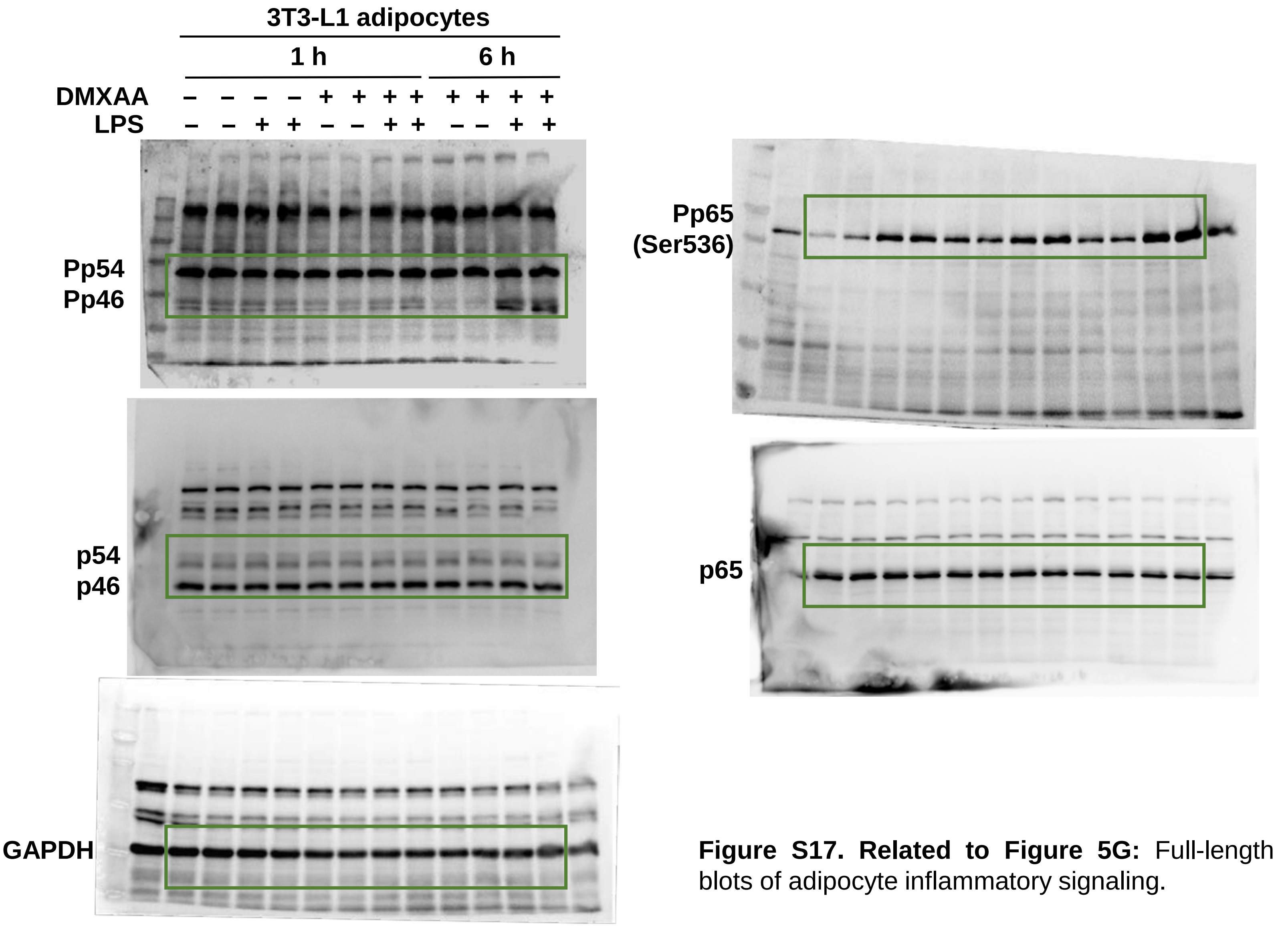

Figure S17. Related to Figure 5G: Full-length blots of adipocyte inflammatory signaling.

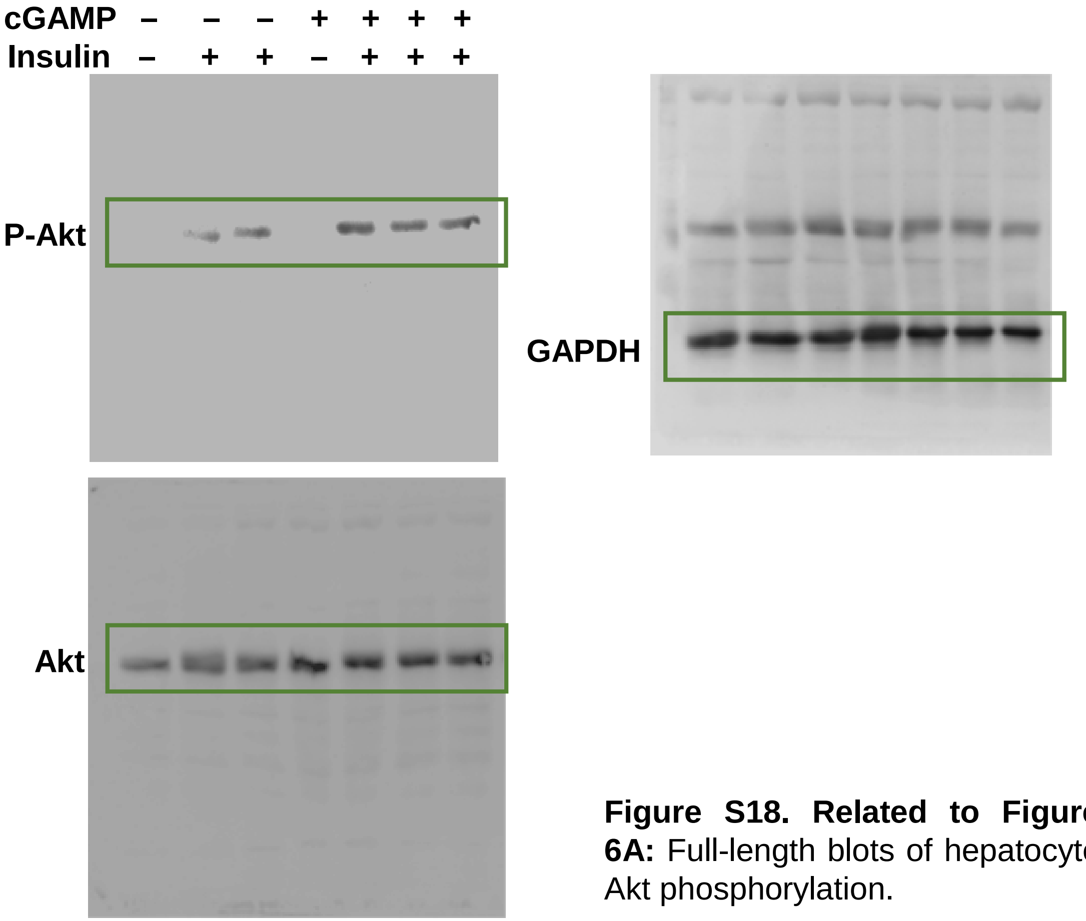

Figure S18. Related to Figure 6A: Full-length blots of hepatocyte Akt phosphorylation.

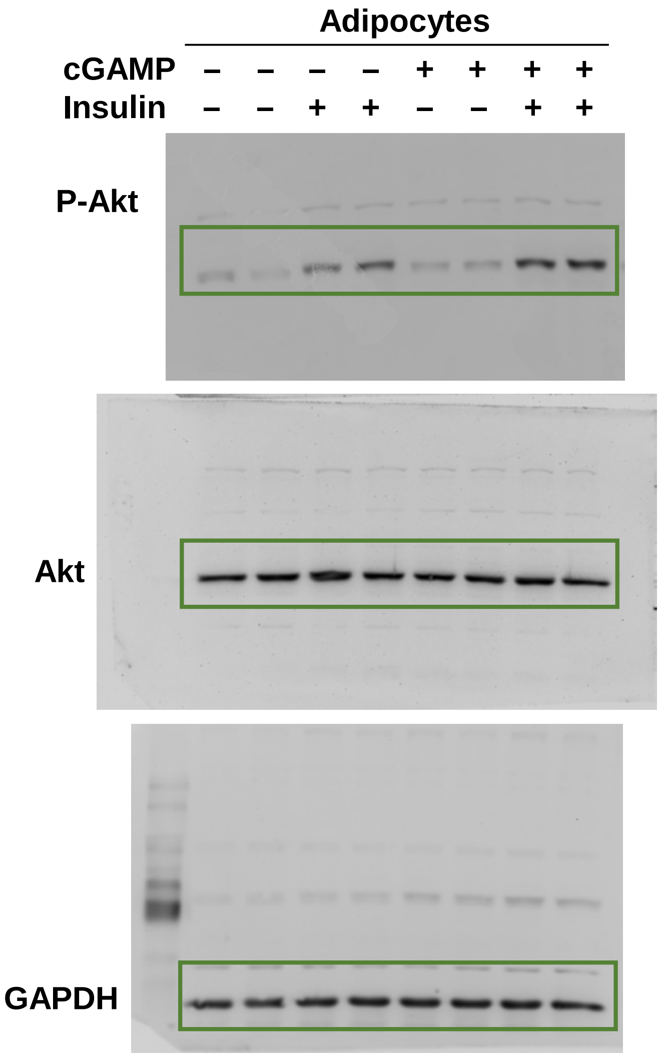

Figure S19. Related to Figure 7A: Full-length blots of adipocyte Akt phosphorylation.
